# Peptide Coacervates with Metal–Phenolic Membranes Modulate Glucose Metabolism and Enhance Cancer Immunotherapy

**DOI:** 10.1002/adma.202510936

**Published:** 2025-09-03

**Authors:** Xin Zheng, Shiqiong Lei, Yiwei Zeng, Lian Chen, Jingqu Chen, Wei Fu, Yu Chen, Xinping Hu, Jing Wang, Meiyuan Jin, Zhichen Liu, Xiaofang Dai, Lei Liu, Wenshan He, Jiajing Zhou, Zhixing Lin, Frank Caruso, Jinghua Ren

**Affiliations:** ^1^ Cancer Center Union Hospital Tongji Medical College Huazhong University of Science and Technology Wuhan 430022 China; ^2^ Hubei Key Laboratory of Precision Radiation Oncology Wuhan 430022 China; ^3^ Department of Breast and Thyroid Surgery Union Hospital Tongji Medical College Huazhong University of Science and Technology Wuhan 430022 China; ^4^ State Key Laboratory of Oral Diseases & National Clinical Research Center for Oral Diseases & Department of Oral and Maxillofacial Surgery West China Hospital of Stomatology Sichuan University Chengdu 610041 China; ^5^ College of Biomass Science and Engineering Key Laboratory of Leather Chemistry and Engineering of Ministry of Education National Engineering Laboratory for Clean Technology of Leather Manufacture Sichuan University Chengdu 610065 China; ^6^ Department of Chemical Engineering The University of Melbourne Parkville Victoria 3010 Australia; ^7^ School of Chemical and Biomedical Engineering Nanyang Technological University 70 Nanyang Drive Singapore 637457 Singapore; ^8^ Shenzhen Huazhong University of Science and Technology Research Institute Shenzhen 518057 China; ^9^ Institute of Radiation Oncology Union Hospital Tongji Medical College Huazhong University of Science and Technology Wuhan 430022 China; ^10^ Present address: Department of Chemical and Petroleum Engineering Khalifa University Abu Dhabi 127788 United Arab Emirates

**Keywords:** combination cancer therapies, memory T cells, metabolic modulation, Metal–phenolic networks, T cell activation

## Abstract

Glucose consumption by tumors induces metabolic restriction of T cells, which results in immune evasion and tumor progression. Regulating cellular metabolism represents a promising strategy to enhance cancer immunotherapy; however, redirecting glucose utilization from tumor cells to T cells is challenging. Herein, the activation of cytotoxic T cells using engineered peptide coacervates (PCs) containing interferon alpha (IFNα) and membranized with metal–phenolic networks (MPNs) (PC‐IFNα@MPNs), which promote glucose uptake and glycolysis, is reported. PC‐IFNα@MPNs modulate the molecular conformation of the co‐stimulatory lymphocyte function‐associated antigen 1 on CD8^+^ T cells, while suppressing tumor cell glycolysis through the sustained release of IFNα, thereby increasing the energy supply for T cells. Furthermore, PC‐IFNα@MPNs suppress tumor progression in preclinical orthotopic tumor mouse models by facilitating T cell infiltration and activation. When combined with immune checkpoint blockade (ICB), PC‐IFNα@MPNs further improve therapeutic outcomes (99% inhibition of tumor growth), even in ICB‐insensitive tumor models. Notably, PC‐IFNα@MPNs exert a robust immune‐memory effect (a 4.8‐fold increase in memory T cells) and provide long‐lasting anti‐tumor activity (over 74 days), thereby preventing postsurgical tumor recurrence. The present study offers insights into metabolic intervention mechanisms mediated by glucose modulation and provides a rational design for metal–organic materials in cancer therapy.

## Graphical abstract

Peptide coacervates encapsulated with interferon‐α and membranized with metal–phenolic networks (PC‐IFNα@MPNs) are designed to reprogram glucose competition between tumor and cytotoxic T cells. This metabolic modulation enhances immunotherapy by suppressing tumor glycolysis and boosting T cell effector function. The combined use of PC‐IFNα@MPNs and surgery prevents tumor recurrence by activating immune memory, offering a promising strategy for cancer therapy.

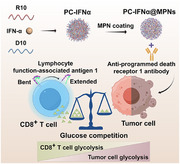

## 1 Introduction

Recent advances in cancer immunotherapy, including immune‐checkpoint blockade (ICB) therapy, adoptive cell transfer therapy, and engineered T cells with chimeric antigen receptors, have revolutionized cancer treatments.^[^ ^1^ ^]^ T lymphocytes, particularly those expressing the cell surface CD8, recognize, attack, and clear tumor cells, playing essential roles in immune surveillance and defense against tumors.^[^ ^1^, ^2^ ^]^ In response to antigenic stimulation, CD8^+^ T cells undergo clonal expansion and differentiate into effector and memory cells,^[^ ^3^ ^]^ where glycolysis is linked to CD8^+^ T cell clonal expansion and effector function.^[^ ^4^ ^]^ However, tumors also sustain malignant growth, proliferation, and metastasis by fueling glycolysis.^[^ ^5^ ^]^ The uptake of glucose by tumor cells restricts nutrient availability for T cell effector function, resulting in a metabolic competitive tumor microenvironment (TME) and immune suppression.^[^ ^6^ ^]^ Concurrently, the glycolytic activity of tumor cells results in the efflux of lactic acid, which adversely affects the anti‐tumor efficacy of T cells.^[^ ^7^ ^]^ Therefore, modulating the metabolic competition between tumors and immune cells is expected to enhance the anti‐tumor efficacy of immune cells and offer therapeutic opportunities to improve immunotherapies.^[^ ^8^ ^]^

Metabolic immunomodulators are considered pivotal agents in cancer therapy, as they can target specific metabolic pathways to increase nutrient availability for immune cells.^[^ ^9^ ^]^ Interferon alpha (IFNα), a pleiotropic cytokine belonging to the type I interferon family, exhibits antiviral and anti‐tumor properties.^[^ ^10^ ^]^ Fan and co‐workers showed that IFNα selectively restricted glucose utilization by tumor cells, thereby establishing a high glucose microenvironment to fuel the activation of CD8^+^ T cells.^[^ ^11^ ^]^ It was also shown that the combined therapeutic effects of IFNα and anti‐programmed death receptor 1 (PD‐1) antibody enhanced immunotherapeutic efficacy in liver cancer. However, subcutaneous administration of IFNα in clinical settings often fails to effectively target tumor tissues and is frequently associated with systemic toxicity, including impairment of renal and hepatic functions.^[^ ^12^ ^]^ Therefore, the development of an IFNα delivery system with targeting ability and combination therapy is a major challenge.

To address this challenge, herein we exploit the adhesive properties of polyphenols, in the form of metal–phenolic networks (MPNs)‐supramolecular materials composed of natural phenolic ligands and metal ions^[^ ^13^, ^14^ ^]^− to encapsulate IFNα. We have previously shown that MPN coatings can act as cytoprotective membranes on peptide coacervates (PCs), facilitating the fabrication of protocells with tailored properties such as controllable permeability and cellular protection.^[^ ^15^ ^]^ Additionally, diverse metal ions (e.g., Fe^3^⁺, Mg^2^⁺), which can serve as immunomodulatory adjuvants to regulate glycometabolic reprogramming and enhance T cell‐mediated anti‐tumor responses,^[^ ^16^ ^]^ can be readily incorporated into MPNs using a generalized protocol.

**Figure 1:**
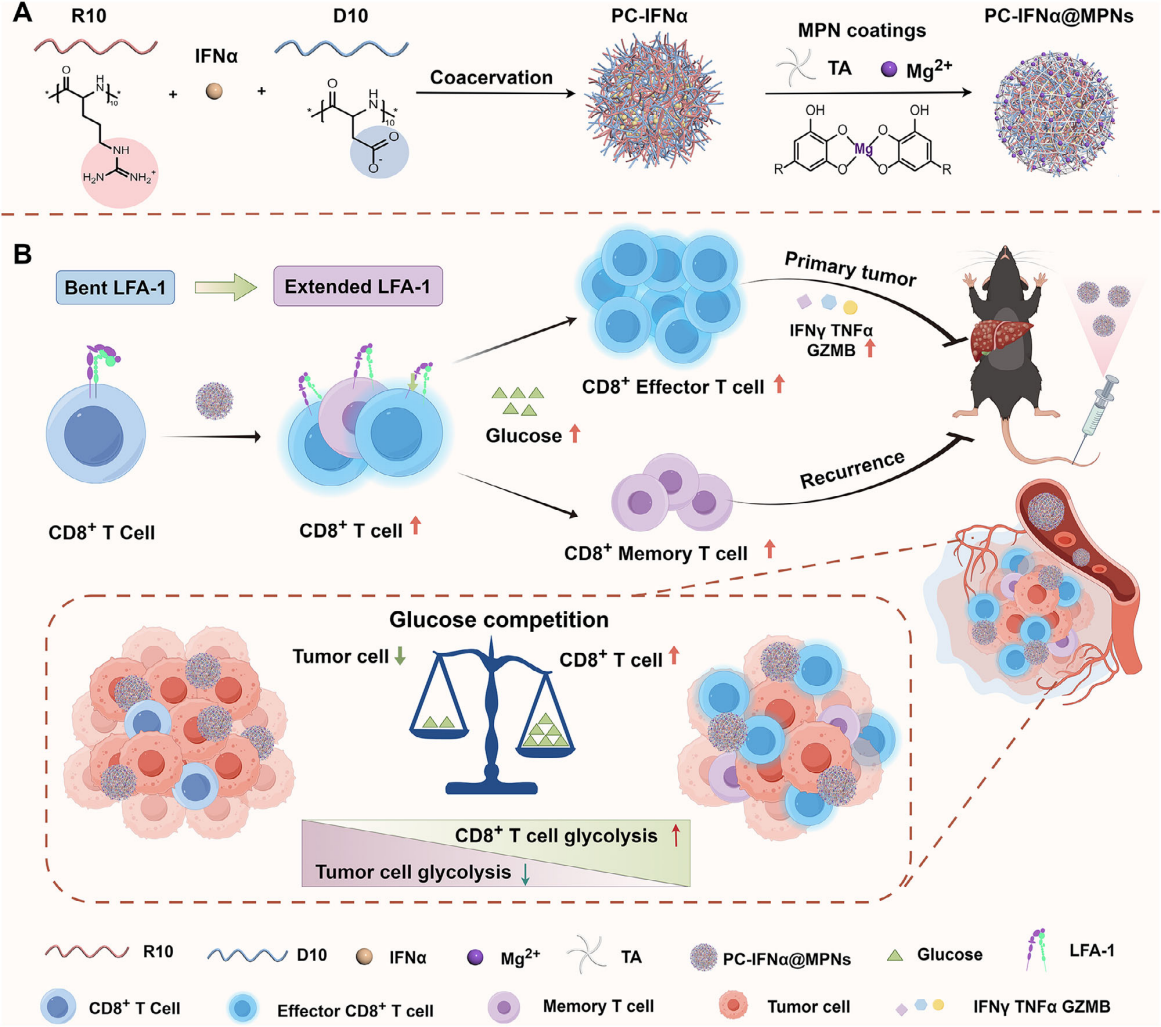
Schematic illustration of the synthesis of PC‐IFNα@MPNs and activation of CD8^+^ T cells. A) Synthesis of PC‐IFNα@MPNs. B) Mechanism of anti‐tumor immune responses induced by PC‐IFNα@MPNs.

Herein, we report Mg^2+^‐based MPN‐coated PCs (PC@MPNs) encapsulating IFNα (PC‐IFNα@MPNs) as a modulator for immunotherapy (**Figure** **1**). PC‐IFNα@MPNs facilitated the proliferation, activation, and specific cytotoxic function in CD8^+^ T cells via mitigating competitive glucose metabolism between tumor and T cells both in vitro and in vivo. The application of both PC‐IFNα@MPNs as a monotherapy and PC‐IFNα@MPNs combined with anti‐PD‐1 antibody as a combined therapy demonstrated biosafety and efficacy in both orthotopic hepatocellular carcinoma (HCC) and breast cancer (BC) mouse models. Importantly, PC‐IFNα@MPNs treatment facilitated the generation of memory T cells in blood and lymphatic tissue, exerting a robust immune‐memory effect and long‐lasting anti‐tumor activity. Furthermore, in the neoadjuvant setting to shrink tumors before surgical excision, PC‐IFNα@MPNs reduced tumor volume and local recurrence after surgical removal of tumors, highlighting its potential as a promising neoadjuvant treatment modality. Our findings suggest that PC‐IFNα@MPNs are a promising platform for ICB combination therapy and neoadjuvant therapy.

## 2 Results and Discussion

### 2.1 Synthesis and Characterization of PC@MPNs and PC‐IFNα@MPNs

**Figure 2:**
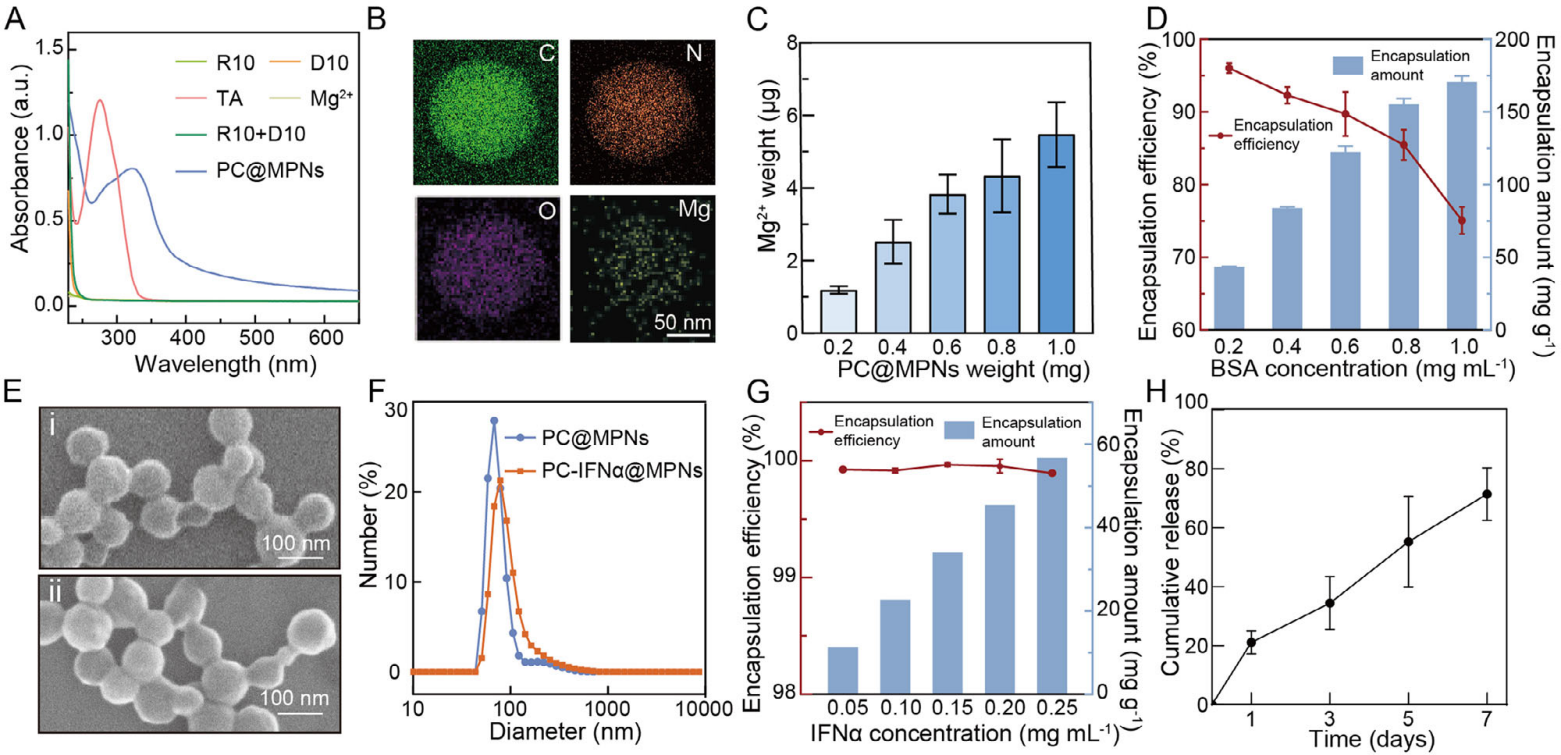
Characterization of PC@MPNs and PC‐IFNα@MPNs. A) UV−vis spectra of material components (R10, D10, TA, Mg^2+^, R10+D10) and PC@MPNs. B) Energy‐dispersive X‐ray spectroscopy elemental mapping of PC@MPNs. C) ICP‐OES analysis of Mg^2+^ content in PC@MPNs. D) Encapsulation amount and efficiency of BSA‐RITC in PC@MPNs determined by fluorescence. E) Scanning electron microscopy images of PC@MPNs (i) and PC‐IFNα@MPNs (ii). F) Size distribution of PC@MPNs and PC‐IFNα@MPNs, as measured by dynamic light scattering. G) Encapsulation amount and efficiency of IFNα in PC@MPNs determined by ELISA. H) Release of IFNα from PC‐IFNα@MPNs in Dulbecco's modified Eagle medium containing 10% FBS determined by ELISA. In (C, D, G, and H), data are presented as the mean ± standard deviation (SD) (*n* = 3).

Building on a previously reported PC@MPNs synthesis protocol,^[^ ^15a^ ^]^ in the present study, we developed a modified approach wherein the aqueous medium is substituted with Tris buffer (7.5 mm, pH 8.5) to achieve improved control over particle size. Specifically, PC@MPNs were synthesized by combining arginine oligopeptides (R10), aspartic acid oligopeptides (D10), with or without cargo, in Tris buffer, followed by the addition of tannic acid (TA) and magnesium chloride (MgCl_2_) to form the coatings (Figure 1A). Substitution with Tris buffer significantly reduced the particle size from ≈650 nm in water to ≈100 nm in Tris buffer (Figure S1, Supporting Information). This is likely because both positively charged R10 (isoelectric point 13.4) and negatively charged D10 (isoelectric point 2.4) are far from their isoelectric points, resulting in stronger net charges and, consequently, enhanced electrostatic interactions between them and smaller particle sizes.^[^ ^15a^ ^]^ The formed particles were then transferred and stored in phosphate‐buffered saline (PBS, pH 7.4) at 4 °C for long‐term storage (Figure S2, Supporting Information) to preserve the stability and bioactivity of the cargo. The successful formation of the resulting nanosized particles was confirmed through various characterization techniques. The UV–vis spectrum of TA showed a characteristic absorption peak at 278 nm, which shifted to 320 nm, indicating the formation of PC@MPNs (**Figure** **2**A). The energy‐dispersive X‐ray spectroscopy element mapping images of PC@MPNs confirmed the uniform distribution of Mg^2+^ in PC@MPNs (Figure 2B). Inductively coupled plasma‐optical emission spectrometry (ICP‐OES) analysis revealed the linear relationship between Mg^2+^ content and PC@MPN concentration, further supporting the incorporation of Mg^2+^ within PC@MPNs (Figure 2C). The stabilizing interactions within PC@MPNs were investigated by incubating PC@MPNs in various competitor solvents. PC@MPNs were almost completely disassembled in Tween 20, indicating the major role of hydrophobic interactions on the stability of PC@MPNs (Figure S3A, Supporting Information). In contrast, partial disassembly was observed after incubation of PC@MPNs in urea, NaCl, ethylenediaminetetraacetic acid, dimethylformamide, or dimethyl sulfoxide, suggesting that hydrogen bonding, ionic interactions, metal coordination, and *π–π* interactions also contribute, though to a lesser extent, to the stabilization of PC@MPNs (Figure S3A, Supporting Information).

To examine the encapsulation capacity of biomacromolecules with varying properties (e.g., molecular weight) in PC@MPNs, rhodamine B isothiocyanate‐labeled bovine serum albumin (BSA‐RITC) was first chosen as the model cargo. Figure S3B (Supporting Information) shows the uniform fluorescence intensity displayed by BSA‐RITC‐encapsulated PC@MPNs, indicating the successful encapsulation of the cargo in PC@MPNs. The encapsulation efficiency of BSA in PC@MPNs was quantified as 96% at a BSA concentration of 0.2 mg mL^−1^ (Figure 2D). Notably, the encapsulation efficiency remained high, for example, 75.1 ± 1.9% at higher BSA concentrations (e.g., 1.0 mg mL^−1^). The encapsulation of IFNα in PC@MPNs was subsequently examined. Scanning electron microscopy and dynamic light scattering analyses revealed the spherical morphology of PC‐IFNα@MPNs (dry state), with diameters of 100 nm, slightly larger than that of PC@MPNs due to cargo loading (Figure 2E,F). The zeta potential of PC‐IFNα@MPNs was −16 ± 1 mV, comparable to that of PC@MPNs (−17 ± 1 mV), indicating that most of the IFNα was encapsulated within the particles (Figure S3C, Supporting Information). Moreover, the Young's moduli of PC@MPNs and PC‐IFNα@MPNs, measured by atomic force microscopy, were ≈2.5 MPa, significantly lower than that of typical MPN coatings (i.e., 2–20 GPa),^[^ ^17^ ^]^ suggesting the soft and liquid‐like nature of the internal coacervates (Figure S3D, Supporting Information). Notably, the Young's modulus of pristine coacervates without coatings is too low to be detected due to their small sizes (≈100 nm) and liquid‐like behavior. The IFNα encapsulation efficiencies of PC@MPNs at IFNα concentrations ranging between 0.05 and 0.25 mg mL^−1^ were >99% (Figure 2G), likely due to the accumulating and aggregation effects of the coacervate phase. Notably, PC‐IFNα@MPNs remained stable in 100% fetal bovine serum (FBS) over an incubation period of 24 h (Figure S3E, Supporting Information). However, the encapsulated IFNα was gradually released in Dulbecco's modified Eagle medium containing 10% FBS over a sustained time period (7 days), likely due to the slow disassembly of the metal–phenolic coordination network by endogenous chelators, such as serum proteins, and reached a cumulative release of 71.5 ± 8.9% over 7 days (Figure 2H).

### 2.2 PC‐IFNα@MPNs Activate CD8^+^ T Cells via Conformational Change of LFA‐1 In Vitro

**Figure 3:**
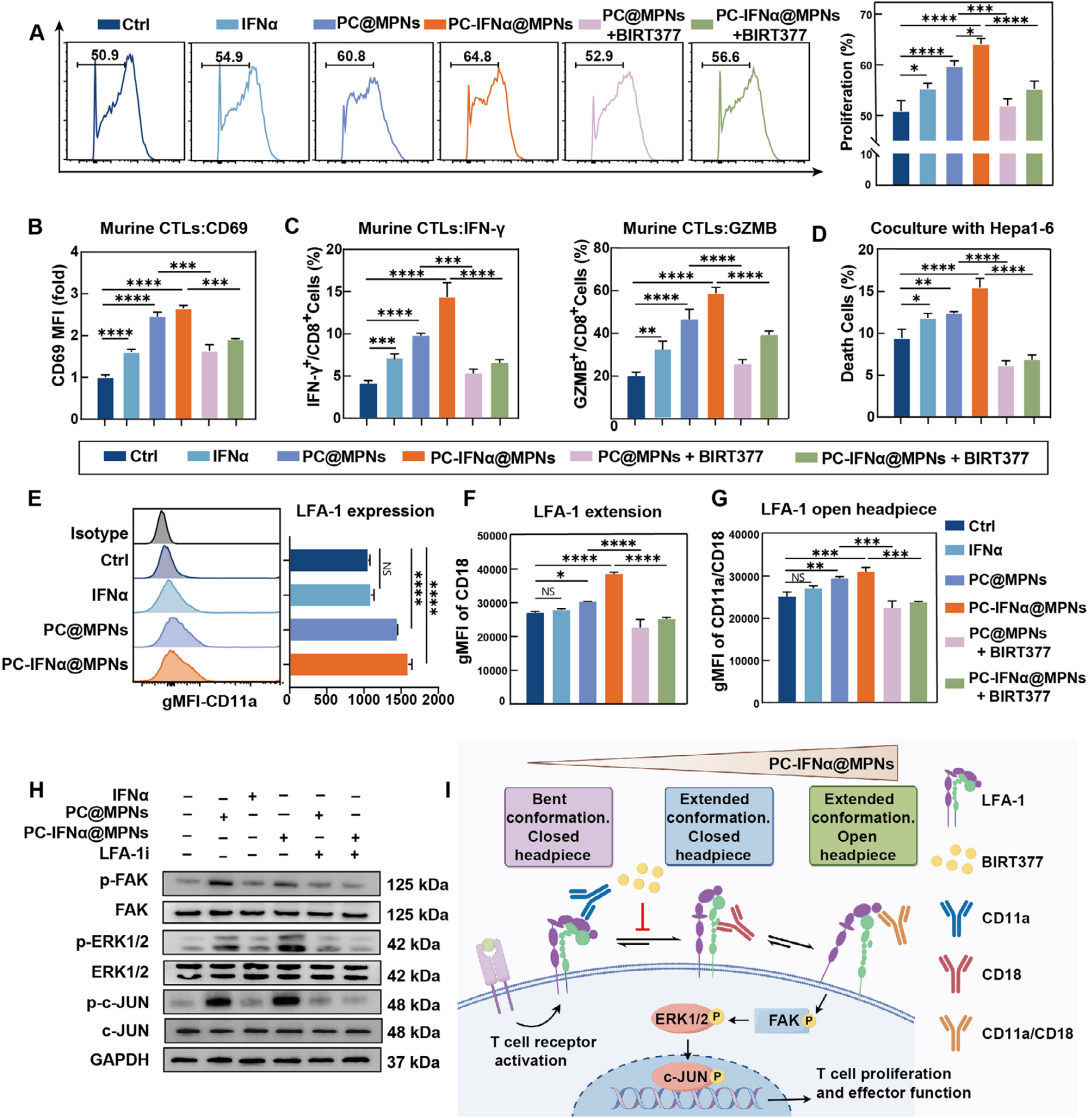
PC‐IFNα@MPNs activate CD8^+^ T cells in vitro via conformational change of LFA‐1. A) Proliferation of CD8^+^ T cells treated with PBS (i.e., Ctrl), IFNα (50 U mL^−1^), PC@MPNs (40 µg mL^−1^), PC‐IFNα@MPNs (40 µg mL^−1^), PC@MPNs+LFA‐1 inhibitor (BIRT377, 10 µm), or PC‐IFNα@MPNs+LFA‐1 inhibitor for 24 h, as measured by CFSE dilution. B,C) Flow cytometry detection of the surface expression of CD69 (B) and the intracellular staining of IFN‐γ^+^ and GZMB^+^ cells in CD8^+^ cells (C). D) Flow cytometry analysis of CFSE‐labeled target Hepa1‐6‐OVA cells cocultured with CD8^+^ T cells, isolated from the spleen of OT‐1 mice, at a CD8^+^ T cell‐to‐Hepa1‐6‐OVA cell ratio of 1:5 under different treatments for 24 h. Samples were stained with 7‐aminoactinomycin D. Cells that stain positive for both 7‐aminoactinomycin D and CFSE represent death target cells. E–G) Flow cytometry analysis of LFA‐1 expression (E), LFA‐1 extended conformation (F), and LFA‐1 open headpiece conformation (G). H) Immunoblot analysis of FAK phosphorylation, ERK1/2 phosphorylation, and c‐Jun phosphorylation in CD8^+^ T cells. I) Schematic illustration of the LFA‐1 conformational state transformation after PC‐IFNα@MPNs treatment and antibodies used to probe them. In (A–G), data are presented as the mean ± SD (*n* = 3). *p*‐values were calculated via one‐way analysis of variance (ANOVA) test. NS, no statistical difference; ^*^ *p* < 0.05; ^**^ *p* < 0.01; ^***^ *p* < 0.001; ^****^ *p* < 0.0001.

Extracellular Mg^2+^, directly sensed by the lymphocyte function‐associated antigen 1 (LFA‐1) molecule on CD8^+^ T cells, is known to boost T cell activation and effector functions by outside‐in activation of focal adhesion kinase (FAK) signaling.^[^ ^18^ ^]^ This process relies on a conformational change in LFA‐1 (extension conformation and headpiece opening), which is stabilized by the binding of Mg^2+^ to metal‐ion‐dependent adhesion sites.^[^ ^19^ ^]^ To examine the regulation of PC‐IFNα@MPNs on CD8^+^ T cell proliferation and effector function in vitro, CD8^+^ T cells activated with CD3/CD28 antibodies were incubated with IFNα (50 U mL^−1^), PC@MPNs (40 µg mL^−1^), or PC‐IFNα@MPNs (40 µg mL^−1^) for 24 h. In line with previous studies that have shown that IFNα can promote T cell proliferation via interferon alpha and beta receptor signaling,^[^ ^20^ ^]^ our findings showed a slight increase in CD8^+^ T cell proliferation from 50.9% in the control group (Ctrl) to 54.9% in the IFNα group by carboxyfluorescein diacetate succinimidyl ester (CFSE) analysis (**Figure** **3**A). The proliferation rates of CD8^+^ T cells in the PC@MPNs and PC‐IFNα@MPNs groups were higher (60.8% and 64.8%, respectively), mainly driven by Mg^2^⁺, with IFNα providing an additional effect.

To investigate the role of the nanoparticles in promoting CD8^+^ T cell proliferation via the active conformation of LFA‐1, we pretreated the cells with BIRT377 (a negative allosteric modulator that stabilizes the bent conformation of LFA‐1)^[^ ^21^ ^]^ before administering PC@MPNs and PC‐IFNα@MPNs treatments. BIRT377 pretreatment effectively inhibited the increase in CD8^+^ T cell proliferation induced by both (i.e., PC@MPNs and PC‐IFNα@MPNs) treatments, indicating that the active conformation of LFA‐1 is essential. To assess the effect of the nanoparticles on CD8^+^ T cell effector function, we cultured CD8^+^ T cells with PC‐IFNα@MPNs for 24 h, which resulted in a 1.65‐fold increase in CD69 expression, an activation marker of leukocytes,^[^ ^22^ ^]^ compared to the control group (Figure 3B). Additionally, we observed an increase in IFN‐γ^+^, TNF‐α^+^, and Granzyme B^+^ (GZMB^+^) CD8^+^ T cells (Figure 3C; Figure S4, Supporting Information). These effects were inhibited upon the administration of BIRT377. To further evaluate the antigen‐specific killing effects of CD8^+^ T cells, we conducted a coculture experiment involving CD8^+^ T cells from ovalbumin (OVA)‐specific OT‐1 mice and tumor cells stably expressing OVA. The target cell killing assay showed an increased killing ability of target Hepa1‐6‐OVA cells by PC‐IFNα@MPNs‐treated OT‐1 murine CD8^+^ T cells (Figure 3D). These findings were also observed in a murine mammary carcinoma 4T1‐OVA cell line (Figure S5, Supporting Information). Consistent with its effects on proliferation and effector activation, BIRT377 treatment also inhibited the antigen‐specific killing capacity previously enhanced by PC@MPNs and PC‐IFNα@MPNs, indicating that the active conformation of LFA‐1 is required for the cytotoxic function of CD8^+^ T cells.

Next, we investigated the conformational changes of LFA‐1 on the surface of CD8^+^ T cells induced by PC‐IFNα@MPNs using conformation‐specific antibodies. Surface expression of LFA‐1 was measured using monoclonal antibody (mAb) CD11a, whereas LFA‐1 extension and headpiece opening were detected using mAb CD18 and mAb CD11a/CD18, respectively. After incubating CD8^+^ T cells with PBS, IFNα, PC@MPNs, and PC‐IFNα@MPNs, we observed increased LFA‐1 expression on CD8^+^ T cells in the PC@MPNs and PC‐IFNα@MPNs groups (Figure 3E). Moreover, LFA‐1 with extension and headpiece opening states were induced in the PC@MPNs and PC‐IFNα@MPNs groups, which could be inhibited by BIRT377 (Figure 3F,G). The active molecular conformation transformation was not observed in the IFNα group, indicating that IFNα had a negligible effect on LFA‐1 regulation. We infer that PC‐IFNα@MPNs enhanced the proliferation, activation, effector function, and specific killing ability of CD8^+^ T cells through increased expression and active molecular conformation of LFA‐1. Previous studies have indicated that the regulation of CD8^+^ T cells by extracellular Mg^2+^ depends on the activation of the mitogen‐activated protein kinase (MAPK) pathway.^[^ ^18^, ^19^, ^23^ ^]^ To determine whether the effects of PC‐IFNα@MPNs are induced by the activation of MAPK signaling in CD8^+^ T cells, we investigated several key effector molecules in the MAPK pathway, including the phosphorylation of focal adhesion kinase (FAK), extracellular regulated protein kinases 1/2 (ERK1/2), and cellular Jun (c‐JUN) proteins. Immunoblot analysis demonstrated that in PC‐IFNα@MPNs, the MAPK signaling pathway was significantly enhanced in CD8^+^ T cells. BIRT377 suppressed the phosphorylation of effector molecules in the MAPK pathway to the levels in the control group (Figure 3H). In summary, Mg^2+^‐based PC‐IFNα@MPNs can modulate the expression and active conformation of LFA‐1, resulting in enhanced proliferation, activation, and effector function of CD8^+^ T cells via MAPK signal transduction (Figure 3I).

### 2.3 PC‐IFNα@MPNs Fuel CD8^+^ T Cells by Limiting Tumor Cell Glycolysis In Vitro

**Figure 4:**
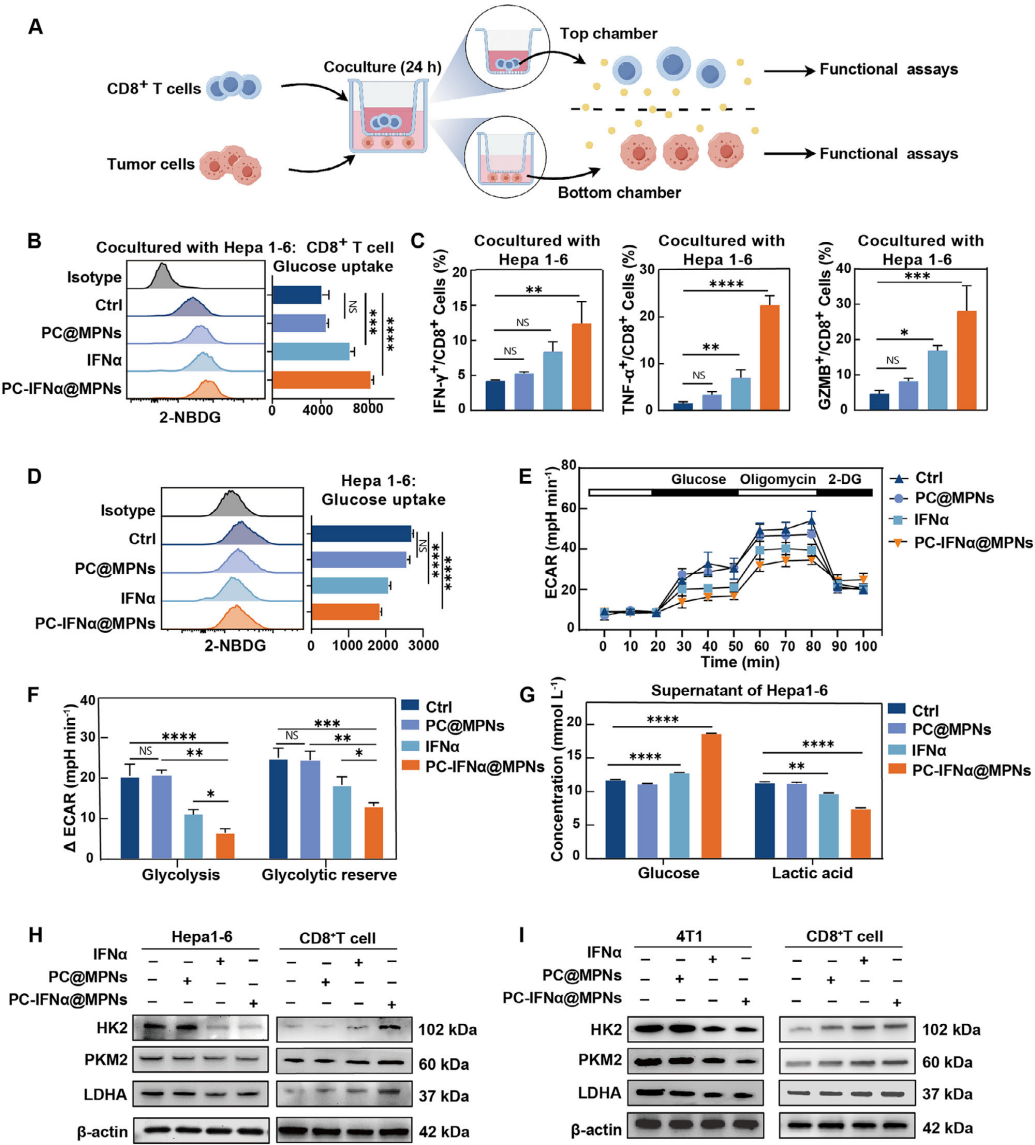
PC‐IFNα@MPNs fuel CD8^+^ T cells via limiting tumor cell glycolysis in vitro. A) Illustration of the coculture system of tumor cells and CD8^+^ T cells. B) 2‐NBDG uptake of CD8^+^ T cells cocultured with Hepa1‐6 after various treatments for 24 h, as evaluated by flow cytometry. C) Intracellular staining of IFN‐γ^+^, TNF‐α^+^, and GZMB^+^ cells in CD8^+^ T cells cocultured with Hepa1‐6. D) 2‐NBDG uptake of Hepa1‐6 in the coculture system. E,F) Assessment of glycolysis and glycolytic reserve of Hepa1‐6 in the coculture system: representative experiment (E) and summary of ECAR (F). G) Concentrations of glucose and lactic acid in the cellular supernatant of Hepa1‐6 after various treatments for 24 h. H,I) Immunoblot assays for HK2, PKM2, and LDHA in tumor cells (Hepa1‐6 or 4T1; left) and CD8^+^ T cells (right) derived from the coculture system. CD8^+^ T cells were cocultured with Hepa1‐6 (H) or 4T1 (I) for 24 h. In (B–G), data are presented as the mean ± SD (*n* = 3). In (B, C, D, F, and G), *p‐values* were calculated via a one‐way ANOVA test. NS, no statistical difference; ^*^ *p* < 0.05; ^**^ *p* < 0.01; ^***^ *p* < 0.001; ^****^ *p* < 0.0001.

Glucose competitive consumption in TME is an essential aspect of cancer metabolism that influences tumor growth and immune response, as glycolysis serves as the central metabolic pathway in T cell activation and tumor progression.^[^ ^24^ ^]^ Targeting the glycolysis pathway in tumor cells to increase glucose concentration and reduce microenvironmental acidosis in TME has emerged as a promising strategy to fuel CD8^+^ T cells.^[^ ^25^ ^]^ As reported elsewhere, IFNα can selectively inhibit glycolysis in tumor cells, which is attributed to the higher interferon alpha and beta receptor expression on tumor cells than on CD8^+^ T cells.^[^ ^11^ ^]^ We hypothesized that PC‐IFNα@MPNs could limit glucose competition between tumor and CD8^+^ T cells. To determine a direct metabolic link between tumor and CD8^+^ T cells, we used an indirect coculture system in vitro via a semi‐permeable membrane in the form of a transwell system, which allows for the exchange of nanoparticles, glucose, lactate, and other low molecular weight proteins (**Figure** **4**A). Flow cytometry assays showed that the glucose uptake by CD8^+^ T cells in the coculture system with Hepa1‐6 increased by almost two fold in the PC‐IFNα@MPNs group relative to the control group (Figure 4B). Flow cytometry analyses further showed that the proportion of CD8^+^ T cells expressing IFNγ, TNFα, and GZMB increased in the PC‐IFNα@MPNs group compared to the IFNα‐treated and control groups (Figure 4C), suggesting a synergistic effect of the Mg^2+^‐based MPNs and the functional component IFNα for T cell activation. Similar results were obtained in CD8^+^ T cells cocultured with the highly glycolytic murine mammary carcinoma 4T1 (Figures S6 and S7, Supporting Information). Furthermore, CD8⁺ T cells cultured alone and treated with PC@MPNs or PC‐IFNα@MPNs also exhibited enhanced glucose uptake, suggesting that IFNα or Mg^2+^ also directly promotes glucose uptake in T cells (Figure S8, Supporting Information). In addition, the glucose uptake of tumor cells Hepa1‐6 and 4T1 was inhibited in the coculture system with PC‐IFNα@MPNs treatment (Figure 4D; Figure S9, Supporting Information).

To further assess the glycolytic capacities of tumor cells, we performed the seahorse extracellular acidification rate (ECAR) assay, which is conventionally used to evaluate glycolysis. The results indicated that the glycolysis levels and glycolytic capacity of Hepa1‐6 cells with PC‐IFNα@MPNs treatment were, respectively, 3.1‐ and 1.9‐fold lower compared with those of the control group (Figure 4E,F). To eliminate the interference of CD8^+^ T cells in the coculture system, tumor cells were cultured alone with PC‐IFNα@MPNs for 24 h to determine their effects on the glucose metabolism of tumor cells. Compared with the control group, the glucose concentration in the Hepa1‐6 and 4T1 cell supernatants increased 1.6 and 2.0 fold, respectively. Furthermore, the level of lactic acid, a product of glycolysis that contributes to the formation of an acidic microenvironment to inhibit the proliferation and activation of immune cells in tumors, was significantly reduced by 34.0% and 19.2% in Hepa1‐6 and 4T1 cells, respectively, relative to the control group (Figure 4G; Figure S10, Supporting Information). These data demonstrate that PC‐IFNα@MPNs restrict glycolysis in tumor cells to increase glucose availability in the coculture system, thereby enhancing CD8^+^ T cell glucose uptake. We also examined messenger RNA and protein levels of genes associated with rate‐limiting glycolytic enzymes, specifically hexokinase2 (HK2), pyruvate kinase M2 (PKM2), and lactate dehydrogenase A (LDHA). These gene levels were upregulated in CD8^+^ T cells but downregulated in tumor cells in the coculture system (Figure S11, Supporting Information; Figure 4H,I). Collectively, these data demonstrate that PC‐IFNα@MPNs selectively inhibit glycolysis in tumor cells and reallocates glucose resources to fuel CD8^+^ T cells, thereby enhancing their effector functions.

### 2.4 PC‐IFNα@MPNs Inhibit Tumor Growth and Improve Anti‐PD‐1 Therapeutic Effect In Vivo

**Figure 5:**
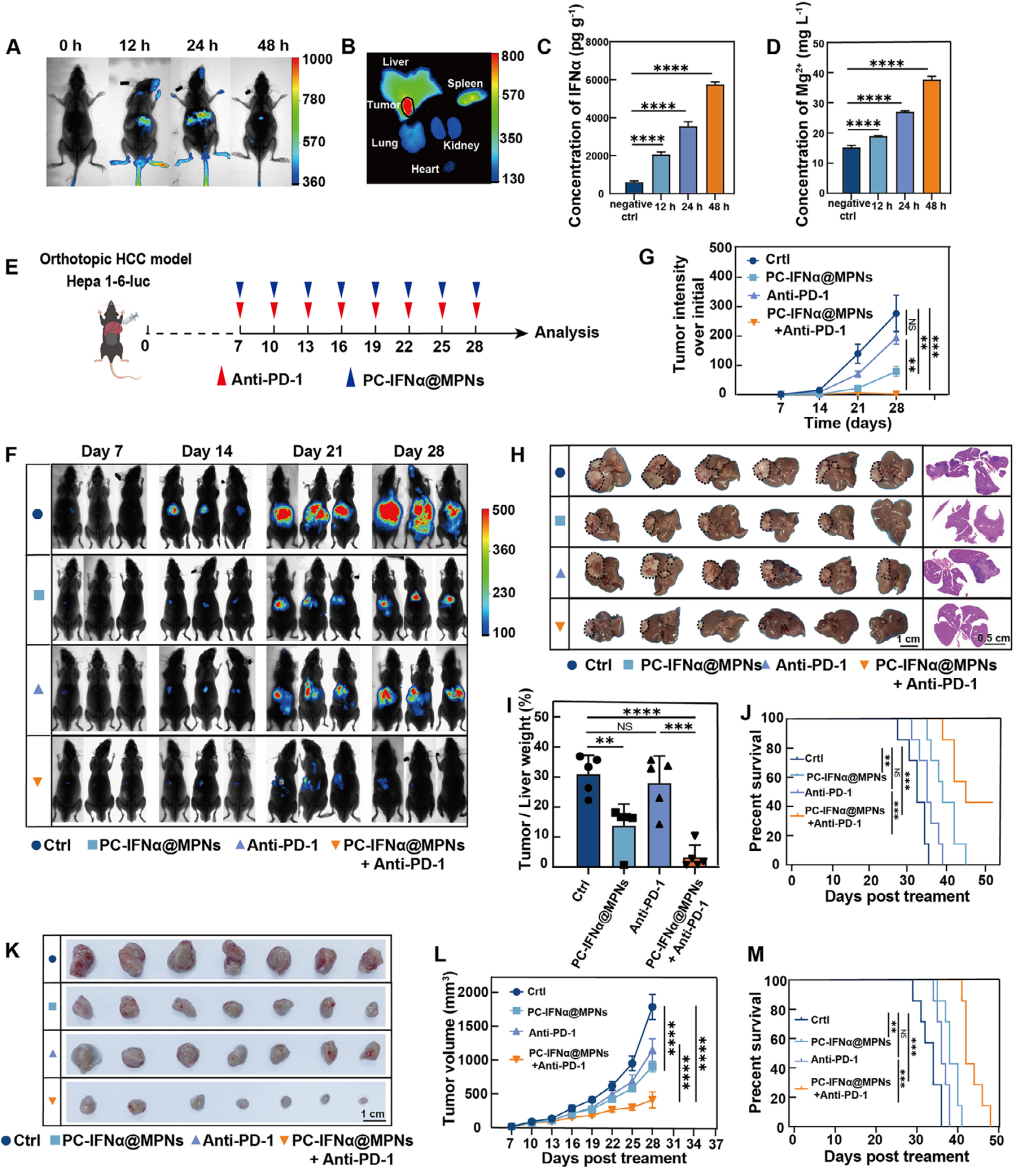
PC‐IFNα@MPNs inhibit tumor growth and improve anti‐PD‐1 therapy in vivo. A) In vivo imaging of mice at different time points after injecting DiR‐labeled PC‐IFNα@MPNs into the tail vein of orthotopic HCC model mice. B) Representative ex vivo fluorescence images of major organs and the primary tumor dissected from mice at 24 h after intravenous injection. with DiR‐labeled PC‐IFNα@MPNs (*n* = 3). C) IFNα levels in tumors from orthotopic HCC model mice after intravenous injection with PC‐IFNα@MPNs (2 mg kg^−1^) (*n* = 3). D) Mg^2+^ levels in tumor tissues from orthotopic HCC model mice after intravenous injection with PC‐IFNα@MPNs via ICP‐MS (2 mg kg^−1^) (*n* = 3). E) Schematic of experimental design of orthotopic HCC model assay. Treatment with PBS (intravenous injection), PC‐IFNα@MPNs (2 mg kg^−1^, intravenous injection), anti‐PD‐1(200 µg mouse^−1^, i.p.), or PC‐IFNα@MPNs+anti‐PD‐1 was performed every 3 days after day 7 of tumor implantation. Mice were euthanized on day 28 post‐tumor implantation (*n* = 6). F) In vivo bioluminescence imaging of Hepa1‐6 tumor growth in orthotopic HCC models subjected to different treatments. Three representative mice per treatment group are shown. Relevant images were taken every 7 days after the model was established. G) Growth curves of tumor bioluminescence in different mouse groups. H) On day 28, after establishing the orthotopic HCC model, the livers from mice subjected to different treatment groups were imaged. I) Bar diagram showing the tumor‐to‐liver weight ratio. J) Survival rate of mice subjected to different treatments as indicated in the orthotopic HCC model (*n* = 6). K) Photographs of tumors collected from mice at day 28 post‐tumor implantation in the orthotopic BC model (*n* = 7). L) Volume growth of tumors in mice subjected to different treatments was monitored over 28 days. M) Survival rate of mice subjected to different treatments as indicated in the orthotopic BC model (*n* = 6). In (C, D, G, I, and L), data are presented as the mean ± SD. *p*‐values were calculated via a one‐way ANOVA test. In (J and M), *p*‐values were calculated via the log‐rank test. NS, no statistical difference; *p < 0.05; **p < 0.01; ***p < 0.001; ****p < 0.0001.

Poor clinical outcomes following ICB therapy, such as anti‐PD‐1 antibody, correlate with the lack of T cell infiltration, T cell exhaustion, and reduced activation in tumors.^[^ ^26^ ^]^ Therefore, combining ICB with immunomodulatory therapy represents an effective strategy to achieve optimal therapeutic outcomes for cancer.^[^ ^27^ ^]^ We hypothesized that PC‐IFNα@MPNs could work synergistically with anti‐PD‐1 antibodies by promoting T cell infiltration and activation in vivo. PC‐IFNα@MPNs, which exhibit a diameter of ≈100 nm, are expected to preferentially accumulate at primary tumor sites and prolong retention in tumor tissues via the enhanced permeability and retention effect.^[^ ^28^ ^]^ To characterize the biodistribution of PC‐IFNα@MPNs, bioluminescence imaging was used, and PC‐IFNα@MPNs were labeled with fluorescent dye DiR in the orthotopic HCC mice (Ethics approval no. ([2023] IACUC number: 4145). As observed from **Figure** **5**A, PC‐IFNα@MPNs were initially enriched around the primary tumor site in mice within 12 h of single intravenous administration; accumulation reached its peak at 24 h post‐injection. The particles were nearly cleared at 48 h post‐injection, while the fluorescence intensities in major organs were at lower levels compared to those of the tumor tissue ex vivo (Figure 5B). Moreover, no hemolysis was observed in mouse blood, even at high concentrations of PC‐IFNα@MPNs in vitro (Figure S12, Supporting Information). These results were further validated in the orthotopic BC mouse model (Figure S13, Supporting Information). Collectively, these results demonstrate the effective tumor‐targeting capabilities and biodegradability of PC‐IFNα@MPNs.

To further assess the drug delivery efficiency of PC‐IFNα@MPNs in vivo, the release of IFNα within tumor tissue was measured using the enzyme‐linked immunosorbent assay (ELISA). The mice received a single dose of PC‐IFNα@MPNs (2 mg kg^−1^), which encapsulated 10^4^ IU IFNα. After intravenous injection of PC‐IFNα@MPNs, the IFNα concentration reached 2063 pg g^−1^ within the tumor tissue at 12 h and continued to increase to 5758 pg g^−1^ at 48 h (Figure 5C). Note that these particles were specifically designed to release IFNα in the tumor interstitial fluid without requiring cellular internalization. Additionally, the concentrations of IFNα in both serum and peritoneum and interstitial fluid of organs (e.g., spleen, lymph node, liver, lung, kidney) were comparatively lower than in tumor tissue (Figure S14, Supporting Information). The increase in IFNα concentration was accompanied by an increase in Mg^2+^ concentration within the tumor tissue, as observed by ICP‐OES (Figure 5D). Overall, the intravenous administration of PC‐IFNα@MPNs efficiently facilitated targeted delivery to primary tumors, thereby mitigating adverse effects.

The intrahepatic tumor model has poor tumor infiltration of immune cells and is unresponsive to anti‐PD‐1 therapy.^[^ ^29^ ^]^ To assess the anti‐tumor efficacy evoked by PC‐IFNα@MPNs in vivo, mice bearing intrahepatic tumors were randomly allocated into four groups. These groups received the following treatments: 1) PBS, 2) PC‐IFNα@MPNs (2 mg kg^−1^) via intravenous injection, 3) mouse anti‐PD‐1 antibody (200 µg mouse^−1^) via intraperitoneal injection (i.p.), and 4) a combination of PC‐IFNα@MPNs with the anti‐PD‐1 antibody administered every 3 days starting from day 7 (Figure 5E). Notably, the particle concentrations were selected to minimize toxicity while maximizing therapeutic efficacy, based on prior studies.^[^ ^11^, ^19^ ^]^ Furthermore, potential chemical interactions between PC‐IFNα@MPNs and the anti‐PD‐1 antibody are negligible due to their distinct administration routes. Bioluminescence imaging was used to measure the tumor bioluminescence signal intensity in vivo. Treatment with PC‐IFNα@MPNs only resulted in an anti‐tumor effect and tumor growth inhibition by 65.7% compared to the control group on day 28. A delay in tumor progression was observed in the anti‐PD‐1 antibody group, but it did not reach statistical significance compared to the control group. Notably, the combination of PC‐IFNα@MPNs and anti‐PD‐1 antibody showed the highest anti‐tumor efficiency (i.e., 99.2%) among all treatment groups (Figure 5F,G). Two of the six mice exhibited a robust anti‐tumor response, with no macroscopically detectable tumors in the liver on day 28 (Figure 5H; Figure S15A, Supporting Information). The average tumor‐to‐liver mass ratio in the mice receiving the combination therapy was only 3.3% compared with 30.9% in the control group (Figure 5I). Additionally, 40% of the mice treated with the combination therapy survived for at least 50 days, indicating that PC‐IFNα@MPNs acted as an immune adjuvant to enhance anti‐PD‐1 efficiency (Figure 5J). In vivo immunotherapy tolerance and side reactions in combination therapies are essential considerations in preclinical safety evaluation.^[^ ^30^ ^]^ Thus, body weight monitoring, serum biochemistry assay, and pathological analysis of major organs were conducted to examine the potential toxicity of our combined therapy. The results revealed no significant impact on body weight (Figure S15B, Supporting Information), hematological parameters (Figure S16A, Supporting Information), or liver and renal function indices (Figure S16B, Supporting Information). Histological examination of major organs, via hematoxylin & eosin staining, revealed no significant differences between the treatment groups (Figure S16C, Supporting Information).

The 4T1 tumor model, characterized by poor immune infiltration and enhanced glycolytic metabolic activity, is relatively resistant to anti‐PD‐1 therapy (Figure S17A, Supporting Information).^[^ ^31^ ^]^ Thus, we assessed whether PC‐IFNα@MPNs could limit glucose metabolism competition to promote anti‐tumor immunity. The combination therapy of PC‐IFNα@MPNs and anti‐PD‐1 antibody displayed the highest therapeutic response and most pronounced tumor growth inhibitory effects among all treatment groups (Figure S17B, Supporting Information; Figure 5K,L). The mice in the combination therapy group showed prolonged survival (48 days) compared with those in the other groups (Figure 5M). These results indicate that PC‐IFNα@MPNs render both checkpoint inhibitor‐resistant murine models responsive to anti‐PD‐1 antibodies.

### 2.5 PC‐IFNα@MPNs Trigger T‐Cell‐Mediated Anti‐Tumor Immune Response and Activation of CD8^+^ Memory T Cells

**Figure 6:**
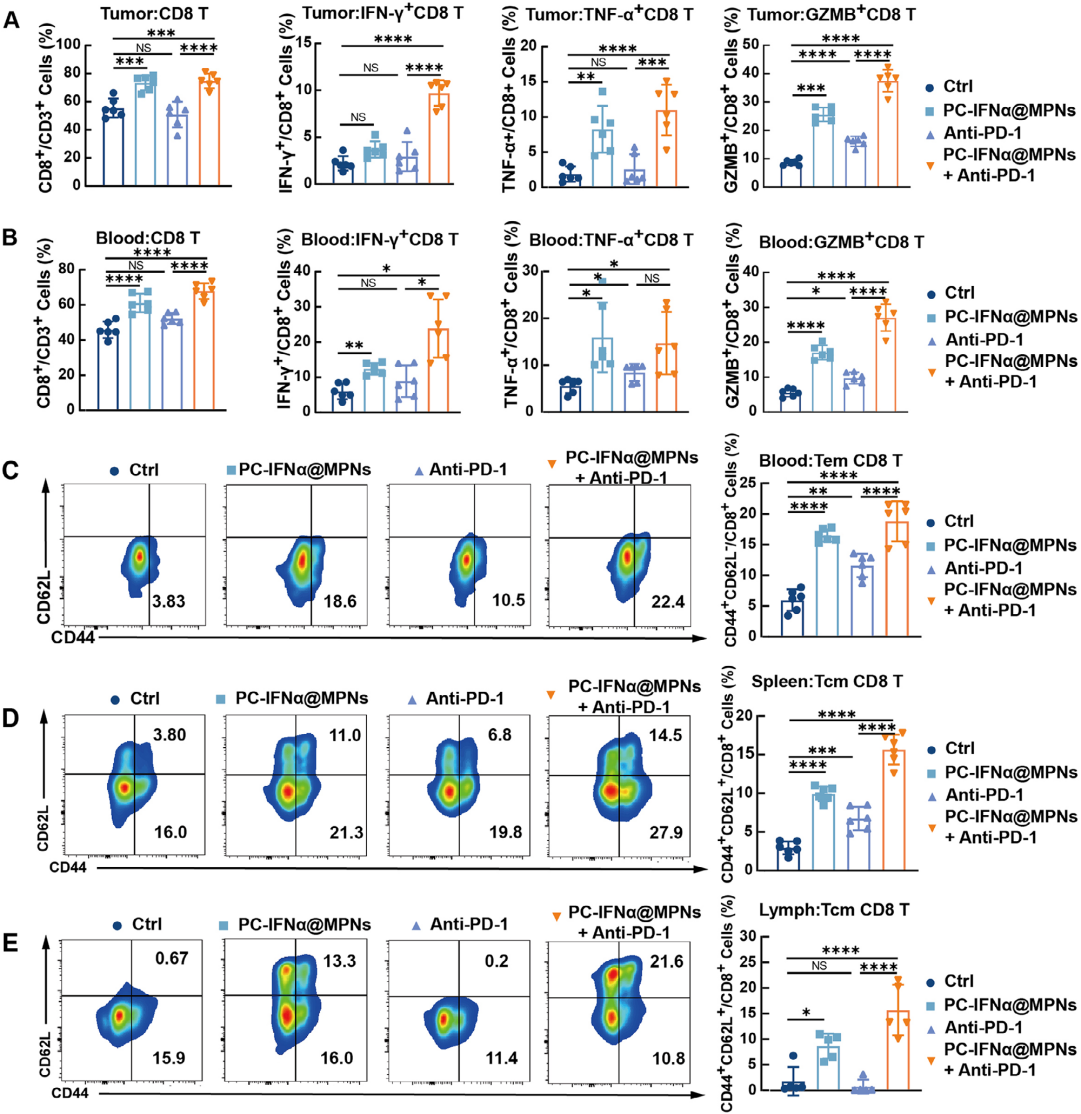
PC‐IFNα@MPNs trigger T‐cell‐mediated anti‐tumor immune response and activate CD8^+^ memory T cells. A,B) Percentages of CD8^+^ T cells, and IFN‐γ^+^, TNF‐α^+^, and GZMB^+^ CD8^+^ T cells in tumor tissue (A) and blood (B) of orthotopic HCC mice model, measured by flow cytometry (*n* = 6). C) Representative flow cytometry analysis images (left) and relative quantification (right) of CD44^+^ CD62L^−^ Tem cell gating on CD8^+^ T cells in peripheral blood of orthotopic HCC mice model (*n* = 6). D,E) Representative flow cytometry analysis images (left) and relative quantification (right) of CD44^+^CD62L^+^ Tcm cells gating on CD8^+^ T cells in the spleen (D) and hepatic lymph nodes (E) of orthotopic HCC mice model (*n* = 5). Data are presented as the mean ± SD. *p*‐values were calculated via a one‐way ANOVA test. NS, no statistical difference; ^*^ *p* < 0.05; ^**^ *p* < 0.01; ^***^ *p* < 0.001; ^****^ *p* < 0.0001.

Next, we further assessed whether PC‐IFNα@MPNs could inhibit tumor growth by enhancing T cell infiltration and activation in vivo. The total number of infiltrating T cells in tumors subjected to various immunotherapeutic regimens was evaluated by flow cytometry. The corresponding gating strategy is illustrated in Figure S18 (Supporting Information). In the orthotopic HCC mouse model, the proportion of CD8^+^ T cells within the tumor increased by ≈17.8% and 19.3% in the combination therapy group and PC‐IFNα@MPNs group, respectively, compared to the PBS group (**Figure** **6**A). The proportion of effector CD8^+^ T cells (IFN‐γ^+^, TNF‐α^+^, and GZMB^+^ CD8 T cells) in tumor tissues, which can secrete cytotoxic factors to kill tumor cells, also increased in both the combination therapy group and PC‐IFNα@MPNs group relative to the control group. These data indicate that PC‐IFNα@MPNs can facilitate the infiltration and effector function of CD8^+^ T cells. Furthermore, the concordant increase in CD8^+^ T cell proportion within peripheral blood and tumor tissue serves as a key prognostic indicator for survival in the context of immunotherapy.^[^ ^32^ ^]^ Flow cytometry analysis revealed an increase in the percentage of CD8^+^ T cells and effector CD8^+^ T cells in the peripheral blood of the combination therapy group, which accounted for the improved survival rates in combination therapy (Figure 6B). These results were further validated in the orthotopic BC model (Figure S19, Supporting Information), where PC‐IFNα@MPNs increased the infiltration and effector function of CD8^+^ T cells, thereby enhancing an anti‐tumor immune response in primary tumors. In addition, the levels of effector memory CD8^+^ T cells (Tem) in peripheral blood, as well as central memory CD8^+^ T cells (Tcm) in the spleen and tumor‐draining lymph nodes, were abundant in both the PC‐IFNα@MPNs group and combination therapy group (Figure 6C–E; Figure S20, Supporting Information), implying robust immune responses in these mice. Tem and Tcm, as the two major circulating memory CD8^+^ T cells subpopulations, are capable of long‐term persistence and functionality across host tissues and tumors, thus providing long‐term invasion resistance to tumor cells.^[^ ^33^ ^]^ In summary, we demonstrated the significant immunotherapeutic efficacy of PC‐IFNα@MPNs and the combination therapy in ICB‐insensitive tumor models, which was attributed to the dual mechanisms of activating anti‐tumor immune responses in CD8^+^ T cells.

### 2.6 Neoadjuvant Therapy Using PC‐IFNα@MPNs Hinder Postsurgical Tumor Regrowth In Vivo

**Figure 7:**
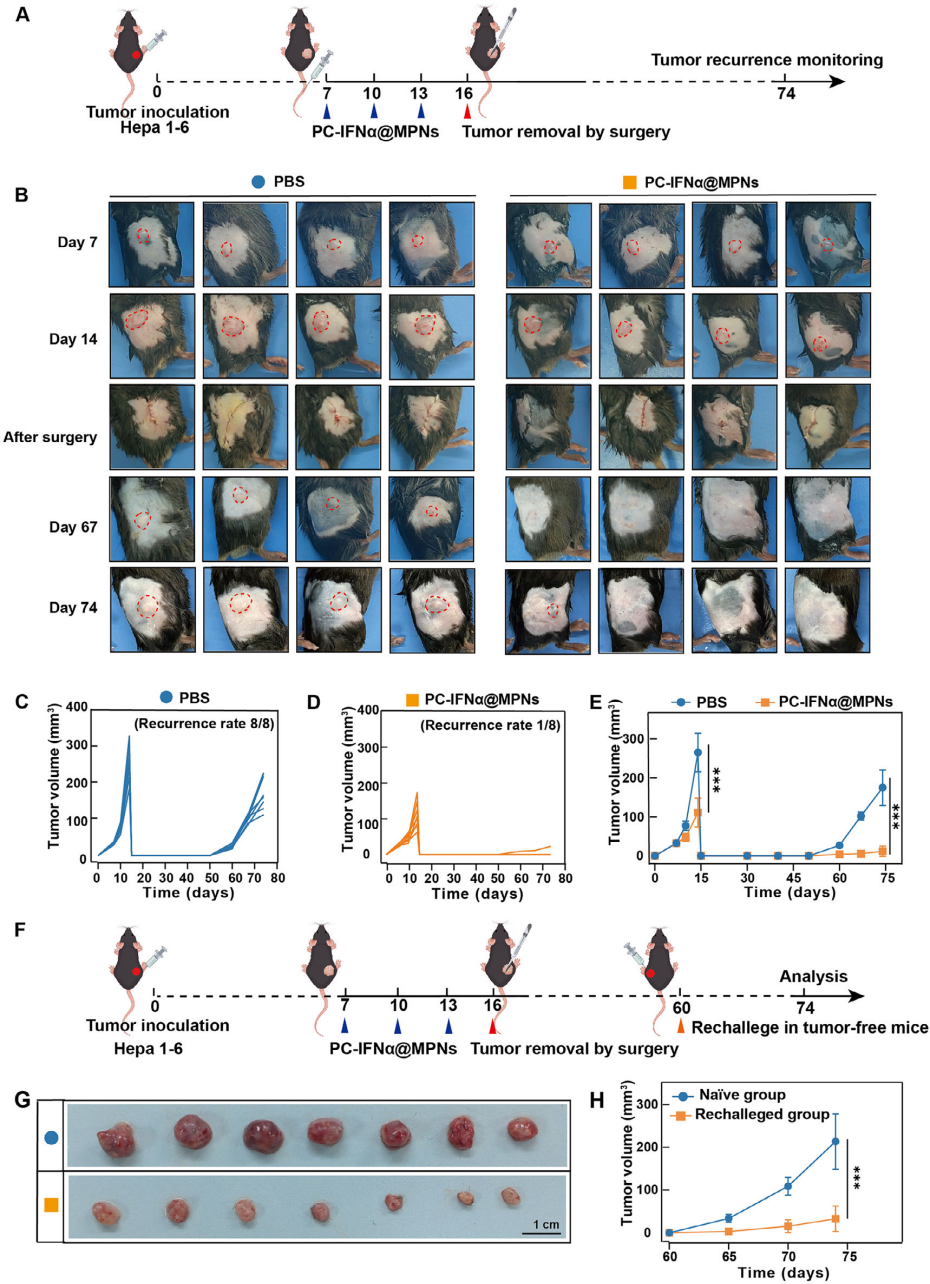
Neoadjuvant therapy using PC‐IFNα@MPNs hinders postsurgical tumor regrowth in vivo. A) Timeline of study conducted using Hepa1‐6 subcutaneous resection tumor model. Intravenous injection of PBS (100 µL mouse^−1^) or PC‐IFNα@MPNs (2 mg kg^−1^) was performed on days 7, 10, and 13 after tumor implantation. Surgical resection of the tumor was conducted on day 16 (*n* = 8). B) Representative photographs of mice following treatments. C,D) Individual primary tumor volume of mice subjected to different treatments. E) Growth curves of primary tumors of mice subjected to different treatments. F) Timeline of the rechallenged tumor mice model. G) Photographs of tumors in the naïve and rechallenged groups. H) Growth curves of the secondary tumor in the naïve and rechallenged groups (*n* = 7). In (E and H), data are presented as the mean ± SD. *p*‐values were calculated via the Tukey test. ^***^ *p* < 0.001.

Memory CD8^+^ T cells can exist in the body for months, even decades; they can launch sustained attacks on tumors and develop enduring anti‐tumor immunity in postsurgical treatment.^[^ ^34^ ^]^ To investigate the long‐term protective effect of PC‐IFNα@MPNs against postsurgical tumor recurrence, we used Hepa1‐6 cells to establish a subcutaneous implantation model in C57BL/6 mice (**Figure** **7**A). To simulate the clinical neoadjuvant therapy of HCC, mice were injected intravenously with PC‐IFNα@MPNs before surgical resection of the tumor. The tumor volume was monitored every 7 days. After three injections of PC‐IFNα@MPNs, tumor growth was suppressed (Figure 7B). These results indicate that the use of neoadjuvant treatment modality PC‐IFNα@MPNs may provide a surgical opportunity for the downstaging of large inoperable malignancies. After surgical tumor resection, tumor growth for each group was recorded (Figure 7C,D). In the surgery‐only group, all mice developed local recurrence on day 74, and tumor volumes reached ≈150 mm^3^. In contrast, there was no local recurrence in over 80% of the mice treated with PC‐IFNα@MPNs (Figure 7E). To explore the versatility of the present neoadjuvant therapy toward other tumor types, we employed the highly invasive 4T1 cells as the BC model (Figure S21A, Supporting Information). Consistent with previous observations, PC‐IFNα@MPNs combined with surgical resection also exhibited therapeutic efficacy against tumor growth and reduced recurrence rates to 62.5% compared to surgical resection alone (Figure S22B–E, Supporting Information). These results demonstrate the use of PC‐IFNα@MPNs as a neoadjuvant approach to promote efficient tumor regression and hinder postsurgical tumor recurrence.

To further assess whether PC‐IFNα@MPNs confer enduring protection against tumor recurrence, we rechallenged tumor‐free mice surviving from a previous survival rate test with Hepa1‐6 cells (1 × 10^7^) contralateral to the primary tumor site on day 60 after tumor resection and treatment (Figure 7F). The naïve mice were also subcutaneously injected with Hepa1‐6 cells in the right flank. All the naïve mice displayed rapid tumor progression, and tumor volumes reached ≈200 mm^3^ at 14 days after tumor implantation, whereas mice that showed no detectable tumors following the combination of PC‐IFNα@MPNs and surgical removal intervention significantly rejected tumor rechallenge, displaying tumor volumes of ≈30 mm^3^ (Figure 7G,H). This therapeutic regimen achieved more than a 6‐fold reduction in tumor burden compared to untreated controls, indicating that PC‐IFNα@MPNs stimulate a long‐lasting immunological memory response to prevent tumor regrowth and represent a promising platform for neoadjuvant therapy in clinical applications.

## 3 Conclusion

We developed a therapeutic tumor‐targeting nanoparticle platform, PC‐IFNα@MPNs, which triggered T‐cell‐mediated anti‐tumor immune response and immune memory through the sustained accumulation of Mg^2+^ and IFNα in TME. This activation in CD8^+^ T cells was attributed to the active conformation of the LFA‐1 molecule and remodeling of glucose competition in TME. In the orthotopic HCC and BC mice models, PC‐IFNα@MPNs promoted the infiltration and function of CD8^+^ T cells in both primary tumors and peripheral blood, effectively inhibiting tumor growth and enhancing anti‐PD‐1 efficiency. Moreover, in the neoadjuvant therapy mouse model, PC‐IFNα@MPNs triggered an immunological memory response and prevented tumor regrowth after surgery. This work provides a rational combination therapy of PC‐IFNα@MPNs and ICB or surgical removal by targeting metabolism to regulate the TME for cancer immunotherapy. Notably, this study demonstrates that the combination of IFNα and Mg^2^⁺ delivered through a simple particle system (i.e., PC‐IFNα@MPNs) can significantly boost systemic memory immunity and be effectively integrated into combination therapy strategies, offering an approach to strengthen long‐term anti‐tumor responses. Moreover, the findings demonstrate the application of MPNs in broadening the use of metabolic modifiers in future ICB and neoadjuvant therapy, providing opportunities for cancer treatment.

## Conflict of Interest

The authors declare no conflict of interest.

## Supporting information



Supporting Information

## Data Availability

The data that support the findings of this study are available from the corresponding author upon reasonable request.
