# Supplementary material for: Peptide Coacervates with Metal–Phenolic Membranes Modulate Glucose Metabolism and Enhance Cancer Immunotherapy
Source: Adv Mater. 2025 Sep 3;37(47):e10936. doi: 10.1002/adma.202510936 (PMC12651129; doi:10.1002/adma.202510936)
Supplement: Supplementary file 1 — Supporting Information [file ADMA-37-e10936-s001.pdf]

ADVANCED MATERIALS

Supporting Information

for *Adv. Mater.*, DOI 10.1002/adma.202510936

Peptide Coacervates with Metal–Phenolic Membranes Modulate Glucose Metabolism and Enhance Cancer Immunotherapy

Xin Zheng, Shiqiong Lei, Yiwei Zeng, Lian Chen, Jingqu Chen, Wei Fu, Yu Chen, Xinping Hu, Jing Wang, Meiyuan Jin, Zhichen Liu, Xiaofang Dai, Lei Liu, Wenshan He, Jiajing Zhou, Zhixing Lin, Frank Caruso* and Jinghua Ren**

Supporting Information

Peptide Coacervates with Metal–Phenolic Membranes Modulate Glucose Metabolism and Enhance Cancer Immunotherapy

Xin Zheng,[+] Shiqiong Lei,[+] Yiwei Zeng,[+] Lian Chen, Jingqu Chen, Wei Fu, Yu Chen, Xinping Hu, Jing Wang, Meiyuan Jin, Zhichen Liu, Xiaofang Dai, Lei Liu, Wenshan He, Jiajing Zhou, Zhixing Lin, Frank Caruso,* and Jinghua Ren**

Table of Contents

Materials and Methods S3
Materials S3
Characterization S3
Synthesis of PC@MPNs S3
Synthesis of PC-BSA @MPNs and PC-IFNα@MPNs S4
Encapsulation Efficiency S4
Release Studies S4
Cell Lines and Cell Culture S4
Murine $CD8^+$ T Cell Isolation and Culture S4
$CD8^+$ T Cell Proliferation and Activation Assays S5
OT-1 T-cell Cytotoxicity Assay S5
Western Blot S5
Metabolic Assays S6
Quantitative Real-Time PCR S6
Animal Studies S6
In Vivo Bioluminescence Imaging S7
Organ Interstitial Fluid Sample Collection S7
Determination of Magnesium Content via ICP-OES S7
In Vitro Cytotoxicity Assay S7
Immunofluorescence Staining S8
Flow Cytometry Analyses S8
Statistical Analysis S8
Supporting Figures S9

Materials and Methods

1. Materials

Tris(hydroxymethyl)aminomethane (Tris), tannic acid (TA), and dimethyl sulfoxide (DMSO) were purchased from Shanghai Aladdin Biochemical Technology Co., Ltd. Magnesium chloride ($MgCl_2$) was obtained from Shanghai Macklin Biochemical Co., Ltd. Oligopeptides of arginine (R10) and aspartic acid (D10) were acquired from Anhui Guoping Pharmaceutical Co., Ltd. Rhodamine B isothiocyanate-labeled bovine serum albumin (BSA-RITC) was purchased from Beijing Solarbio Science & Technology Co., Ltd. Mouse interferon alpha (IFNα) was purchased from Miltenyi Biotec. DiR iodide was purchased from MedChemExpress. Phosphate-buffered saline (PBS) and mouse IFNα enzyme-linked immunosorbent assay (ELISA) kit was purchased from Thermo Fisher Scientific. A Millipore water purification system provided high-purity water with a resistivity of 18.2 MΩ cm.

2. Characterization

2.1. Scanning electron microscopy images were captured on a Phenom Pharos G2 microscope (Thermo Fisher Scientific), operating at 15 kV.
2.2. Energy-dispersive X-ray spectroscopy profiles were acquired with a Thermo Fisher Talos F200S device, functioning at 200 kV.
2.3. UV–Vis absorption and fluorescence spectra were recorded using a BioTek Synergy H1 instrument.
2.4. Dynamic light scattering measurements were performed using a Malvern NANO-ZSP Zetasizer.
2.5. Bright-field and fluorescence images were captured on an Olympus IX73 inverted fluorescence microscope.
2.6. Mg^{2+} concentrations were measured by inductively coupled plasma-optical emission spectrometry.

3. Synthesis of PC@MPNs

R10 was dissolved in water at a concentration of 1 mg mL^{-1}. Separately, D10 was dissolved in DMSO at a concentration of 10 mg mL^{-1} and then diluted in Tris buffer (5 mM, pH 8.5) to achieve a final concentration of 1 mg mL^{-1}. The coacervates formed upon mixing R10 and D10 in Tris buffer (7.5 mM, pH 8.5) at an R10-to-D10 ratio of 1:3 (v/v). This mixture was supplemented with TA (10 µL, 1.0 mg mL^{-1}) and $MgCl_2$ (20 µL, 3.7 mM). The pH of the suspension was raised by adding Tris buffer (30 µL, 10 mM, pH 8.5). After incubation for 10 s, the PC@MPN particles were collected by centrifugation (10000 *g*, 3 min) and washed with water twice. The PC@MPN dispersion was stored at 4 °C prior to use.

4. Synthesis of PC-BSA@MPNs and PC-IFNα@MPNs

To encapsulate BSA-RITC or IFNα into PC@MPNs, BSA-RITC (10 μL) or IFNα (10 μL) was mixed with R10 (10 μL) and D10 to form cargo-encapsulated coacervates before the successive addition of TA and $MgCl_2$.

5. Encapsulation Efficiency

To quantify the encapsulation efficiencies of BSA-RITC and IFNα in PC@MPNs, BSA-RITC (10 μL; 0.2, 0.4, 0.6, 0.8, and 1.0 mg mL^{-1}) or IFNα (10 μL; 0.05, 0.10, 0.15, 0.20, and 0.25 mg mL^{-1}) was individually mixed with R10 (10 μL) and D10 before the successive addition of TA and $MgCl_2$. The encapsulation efficiency in PC@MPNs was determined by subtracting the unencapsulated amount from the total quantity of added cargo. Fluorescence intensity measurements at 600 nm, with an excitation wavelength of 560 nm, were performed on an equivalent volume of supernatant obtained from the initial centrifugation step to determine the unencapsulated amount of BSA-RITC. The unencapsulated amount of IFNα was measured using a mouse IFNα ELISA kit.

6. Release Studies

To evaluate the release of IFNα from PC-IFNα@MPNs, samples were immersed in Dulbecco's modified Eagle medium containing 10% fetal bovine serum (FBS) at 37 °C for 7 days. The supernatants were collected on days 1, 3, 5, and 7, and the release of IFNα was evaluated by ELISA.

7. Cell Lines and Cell Culture

The murine Hepa1-6 cell line and murine 4T1 cell line were purchased from the American Tissue Culture Collection. Luciferase-stably transfected cell lines (hepa1-6-luc) were inoculated in mouse livers to monitor the tumor growth rate through bioluminescence imaging. These cells were cultured in Dulbecco's modified Eagle medium (Gibco, USA) or Roswell Park Memorial Institute (RPMI) 1640 (Gibco, USA) supplemented with 10% FBS (Gibco, USA) and antibiotics (penicillin and streptomycin; Biosharp, China) at 37 °C in 5% CO_2. Routine tests for mycoplasma infection were conducted on all cell lines, and they were confirmed to be negative. Immune cell culture is described in Section 8. Hepa1-6-luc cells that were stably transfected with firefly luciferase were established for in vivo assays.

8. Murine CD8$^+$ T Cell Isolation and Culture

The naïve CD8$^+$ T cells from the spleens of C57BL/6 mice were magnetically isolated using a MojoSort Mouse CD8$^+$ T Cell Isolation Kit (BioLegend, USA). Naïve CD8$^+$ T cells were activated on a 24-well plate pre-processed with anti-CD3 Ab (BioLegend, USA) and anti-CD28 Ab (BioLegend, USA). CD8$^+$ T cells were then cultured in RPMI-1640 medium supplemented with 10% FBS, 10 ng mL^{-1} interleukin-2 (BioLegend, USA), and 27.5 μM 2-mercaptoethanol at 37 °C in 5% CO_2 for subsequent experiments.

9. CD8$^+$ T Cell Proliferation and Activation Assays

To analyze cell proliferation, carboxyfluorescein diacetate succinimidyl ester (CFSE, BioLegend, USA) was used to prelabel murine CD8$^+$ T cells. CD8$^+$ T cells were then cultured with specified treatments for 72 h at 5 × 10^4 cells per well on a 96-well plate. For evaluation of surface marker and intracellular cytokine staining, CD8$^+$ T cells were cultured with specified treatments on a 96-well plate for 24 h at 5 × 10^4 cells, washed once. Cells were incubated with specific antibodies for 20 min at 4 °C, followed by washing with permeabilization buffer (BioLegend, USA) before intracellular cytokine staining. To analyze the expression and activation conformation of LFA-1, anti-mouse CD11a, CD18, and CD11a/CD18 (BioLegend, USA) were used to probe expression, extension, and open headpiece of LFA-1. The cells were incubated with antibodies on ice for 30 min. All cells were washed twice in fluorescence-activated cell sorting (FACS) buffer before detection.

10. OT-1 T-cell Cytotoxicity Assay

Tumor cells with ovalbumin (OVA) were first labeled with 10 μM CFSE and then cocultured with splenic OT-I CD8$^+$ T cells at a CD8$^+$ T cell-to-Hepa1-6-OVA cell ratio of 1:5. The cells were then subjected to different treatments for 24 h before tumor cell death analysis. To identify tumor cell death, the supernatant cells were collected and then incubated (stained) with 7-aminoactinomycin D (7-AAD) fluorescent probe (BioLegend, USA), followed by via flow cytometry analysis. Cells that stain positive for both 7-AAD and CFSE represent death target cells.

11. Western Blot

Cells were lysed by using radioimmunoprecipitation assay (RIPA) buffer (MCE, USA) with protease and phosphatase inhibitors (MCE, USA), and protein concentrations were assessed with a BCA protein assay kit (MCE, USA). After heating at 100 ℃ for 8 min to denature the cells, whole-cell lysates were separated via 4–20% sodium dodecyl sulfate-polyacrylamide gel electrophoresis (Epizyme, China) and transferred onto polyvinylidene fluoride membranes. The membranes were incubated with these primary antibodies: FAK (Cell Signaling Technology, #3285; 1:1000), p-FAK (Cell Signaling Technology, #3283; 1:1000), p44/42 MAPK (Erk1/2) (Cell Signaling Technology, #137F5; 1:1000), phospho-p44/42 MAPK (Erk1/2) (Thr202/Tyr204) (Cell Signaling Technology, #71433T; 1:1000), c-JUN (Cell Signaling Technology, #60A8; 1:1000), Phospho-c-Jun (Ser73) (Cell Signaling Technology, #3270; 1:1000), HK2 (Cell Signaling Technology, #2267; 1:1000), PKM2 (Cell Signaling Technology, #4053; 1:1000), LDHA (Cell Signaling Technology, 3582; 1:1000), GAPDH (Cell Signaling Technology, #2218; 1:1000), and β-actin (Cell Signaling Technology, #4970; 1:1000). The blots were subsequently stained using horseradish peroxidase-conjugated secondary antibodies against rabbit or mouse (MCE, USA). The protein was visible using an Ultra High Sensitivity ECL Kit (MCE, USA).

12. Metabolic Assays

For the glucose uptake studies, 2-NBDG was used to evaluate cell glucose uptake. After various treatments, cells were incubated with 2-NBDG (final concentration of 50 μM, Selleck, USA) for 1 h. Cells were washed twice in FACS buffer before detection. For the glucose and lactate assays, the supernatant of tumor cells in 6-well plate was collected, and the glucose and lactate concentrations were assessed using Glucose Quantification Kit and L-Lactate Assay (Beyotime Biotechnology, China). For extracellular acidification rate assays, real-time extracellular acidification rate was measured on an XFe24 extracellular flux analyzer (Seahorse Bioscience, Agilent, USA).

13. Quantitative Real-Time Polymerase Chain Reaction (qPCR)

A Total RNA Kit I (Omega, USA) was used to extract total RNA from cells and was then employed as a template to produce cDNA with the ReverTra Ace® qPCR RT Master Mix (Vazyme, China). Quantitative real-time PCR was performed using ChamQ Universal SYBR qPCR Master Mix (Vazyme, China) and analysis was performed using StepOne Software v2.3. The specific primers were shown as follows: HIF-1α, forward 5′-GCTAAGGCATCAGCATAC-3′, reverse 5′-GCTATTGTCTTTGGGTTT-3′; PKM2, forward 5′-GCAGGAGTGCTCACCAAG-3′, reverse 5′-GCCAAGTTTACACGAAGG-3′; LDHA, forward 5′-ACAGTTGTTGGGGTTGGT-3′, reverse 5′-ATCTCGCCCTTGAGTTTG-3′; HK2, forward 5′-TACACTCAATGACATCCGAACT-3′, reverse 5′-TCCGTCCTTATCGTCTTCAA-3′; GLUT1, forward 5′-ATCCTGTTGCCCTTCTGC-3′, reverse 5′-AGGTCTCGGGTCACATCG-3′; GAPDH, forward 5′-GGTGAAGGTCGGTGTGACCG-3′, reverse 5′-CTCGCTCCTGGAAGATGGTG-3′.

14. Animal Studies

Female BALB/c and male C57BL/6J mice (6 weeks old) were purchased from the Vital River Laboratory Animal Technology Co. (Beijing, China) and kept in neutral housing conditions at Tongji Medical College Animal Experimentation. The experiments were conducted according to the guidelines and received ethical approval from the Ethics Committee of Tongji Medical College, HUST, Wuhan, China ([2023] IACUC number: 4145). For orthotopic hepatocellular carcinoma (HCC) models, C57BL/6J mice were implanted into the left lobe of the liver parenchyma with 1×10^6 Hepa1-6-luc cells in PBS (20 μL). For orthotopic breast cancer (BC) models, female BALB/c mice were implanted into the right breast pad with 2×10^4 4T1 cells in PBS (50 μL). After implanting for 7 days, mice bearing Hepa1-6-luc or 4T1 orthotopic tumors were randomly sorted into the indicated groups. For combination therapy, mice were treated intravenously with PC-IFNα@MPNs (2 mg kg^{-1}) and/or anti-programmed death receptor 1 (anti-PD-1) antibody (10 mg kg^{-1}) (BE0146, Bio X Cell, USA) following the timeline in Figure 5E. Tumor growth was monitored by bioluminescence imaging or measured using a vernier caliper every 3–5 days. Images were analyzed to calculate the volume of the tumors using Bruker In Vivo MS FX PRO analysis software. Tumor volume was calculated as follows: length (mm) × $width^2$ (mm^2) × 0.5. Mouse body weight was routinely monitored. All studies that applied anesthesia or euthanasia

methods complied with the generally accepted veterinary best practices. For subcutaneous complete surgical tumor resection mice model, PBS (50 μL) with Hepa1-6 cells (1×10^6) or 4T1 cells (2×10^4) were subcutaneously injected into the right flank of C57BL/6 or BALB/c mice. After inoculation for 7 days, the mice were randomized into two groups (8 mice per group). Intravenous injections were performed every 3 days, with a total of 6 treatments. Tumors were then resected completely after 3 intravenous administrations. For the rechallenged mice model, the even-aged naïve C57BL/6 mice and curative mice in the PC-IFNα@MPNs and surgical resection mice were transplanted into the left flank of mice with Hepa1-6 cells (1×10^5). After 14 days, the mice were euthanized, and the tumors were resected from mice. All schematics were prepared using Figdraw.

15. In Vivo Bioluminescence Imaging

The orthotopic HCC and BC models were established as described in Section 14. On day 7, DiR-labeled PC-IFNα@MPNs (100 μL) were injected into tumor-bearing mice via tail vein; the mice were examined at 12, 24, and 48 h under 750 nm excitation light on a Bruker In Vivo MS FX PRO instrument to obtain spectrum images of the distribution of nanoparticles in vivo. The distribution of DiR-labeled PC-IFNα@MPNs in the organs harvested from the mice was analyzed 24 h after drug injection.

16. Organ Interstitial Fluid Sample Collection

After intravenous injection with PC-IFNα@MPNs for 12, 24, and 48 h, the tumor, liver, spleen, kidney, and tumor-draining lymph nodes (hepatic lymph nodes of orthotopic HCC model and inguinal lymph nodes of orthotopic BC model) were aseptically removed. The tissue (0.1g) of various organs was mixed with PBS (1 mL) and centrifuged at 300 *g* for 10 min at 4 °C. Peritoneal fluid was collected from the lavage after injecting sterile PBS (1 mL) into the peritoneal cavity and gently massaging. The supernatants of various organs were stored at −80 °C. The concentrations of IFNα in tumor and organ supernatants were determined using the mouse IFNα ELISA kit.

17. Determination of Magnesium Content via Inductively Coupled Plasma–Optical Emission Spectrometry (ICP-OES)

An aliquot (500 μL) of the samples was added to HNO_3 (6 mL, 67–69%) and heated at 95 °C for 2 h. Digestates were filled up to 5 mL volume using dd-H_2O. The samples were analyzed using ICP-OES (PerkinElmer, USA) on an 8800 system (Agilent, Switzerland) with standard settings.

18. In Vitro Cytotoxicity Assay

A density of 5×10^3 LO-2 cells per well was used to inoculate 96-well plates and then incubated with PC-IFNα@MPNs at concentrations ranging from 0 to 150 μg mL^{-1} for 24 h. The effect of PC-IFNα@MPNs treatment on cell proliferation was determined using the CCK8 assay (Biosharp, China). Calcein-AM/PI staining was used to differentiate between LO-2 cells that survived or died after treatments. After removing

the medium, cells underwent gentle wash with PBS and then stained with a calcein-AM and PI solution mix for 20 min. Cells were imaged via fluorescence microscopy.

19. Immunofluorescence Staining

Tumor cells were grown exponentially on 12-well plates that contained pre-placed coverslips and cultured with DiR-labeled PC-IFNα@MPNs medium (40 μg mL^{-1}) for 24 h. The medium was taken out and rinsed with PBS three times. Subsequently, the tumor cells were treated with 4% paraformaldehyde for 20 min at room temperature, followed by permeabilization with 0.1% Triton X-100 in PBS for 5 min and staining with fluorescein isothiocyanate–phalloidin for 20 min and 4′,6-diamidino-2-phenylindole for 5 min. Before introducing each new reagent, the cells were washed thrice with PBS. Cells were imaged using a laser confocal microscope (Nikon, Japan).

20. Flow Cytometry Analyses

For flow cytometry analysis of murine blood, tumor-draining lymph nodes, spleens, and tumors, mice were anesthetized with pentobarbital sodium; blood was collected by cardiac bleeding. Tumor-draining lymph nodes and spleens were mechanically dissociated to generate single-cell suspensions, which were then filtered through a 40 μm mesh. Tumor tissues were decomposed using a buffer with 0.2% collagenase V, 0.01% hyaluronidase, and 0.002% DNase I at 37 °C for 1 h and then filtered through a 40 μm mesh. Erythrocytes were lysed using a lysis buffer (Biosharp, China). After counting the cells (1×10^6), the cells were firstly incubated with Fc block (BioLegend, USA) for 20 min, incubated with fixable live/dead stain for 30 min, and then incubated with fluorochrome-conjugated antibodies against the following mouse antigens: APC-CD45, APC-cy7-CD33, BV650-CD4, PE-CD8, BV605-CD62L, PerCP/Cy5.5-CD44, BV421-IFNγ, BV785-TNFα, and PE/Dazzle 594-GZMB. Flow cytometry analysis was performed using a BD FACSCelesta instrument, and all FACS data were analyzed and representative flow plots were generated by FlowJo software (TreeStar, Ashland, USA).

21. Statistical Analysis

At least three repetitions were done for all experiments. Data were expressed as mean ± standard deviation and analyzed statistically using GraphPad Prism 9 software. Sample size (n) for each statistical analysis has been indicated in the legend of the corresponding figures. The statistical differences between the two groups were evaluated using a one-way analysis of variance and a Tukey test. Survival analysis employed the log-rank test, considering a p-value of less than 0.05 as significant. An asterisk (*) denotes statistically significant difference between the respective bars (NS, no statistical difference; *, $p < 0.05$; **, $p < 0.01$; ***, $p < 0.001$; ****, $p < 0.0001$).

Supporting Figures

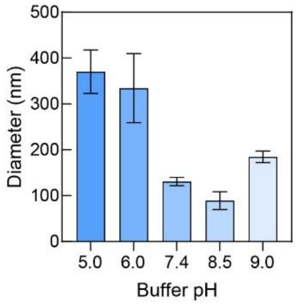

Figure S1. Size distribution of PC@MPNs synthesized in buffers of different pH, measured by dynamic light scattering.

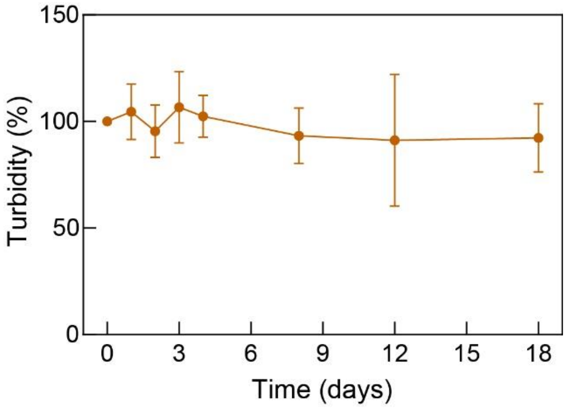

Figure S2. Turbidity measurements of PC@MPNs stored in PBS at 4 °C over 18 days.

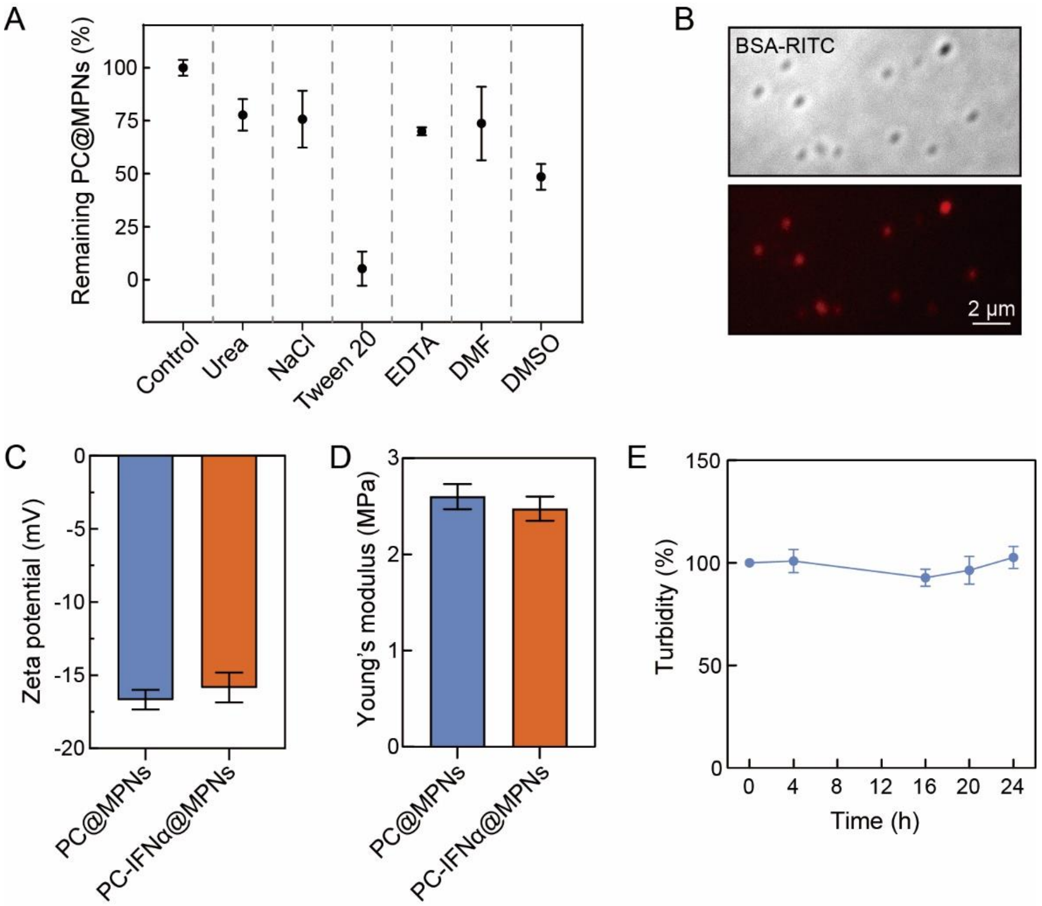

Figure S3. (A) Stability of PC@MPNs in different media (urea, NaCl, Tween20, ethylenediaminetetraacetic acid (EDTA), dimethylformamide (DMF), and DMSO). Stability was evaluated by measuring turbidity (absorbance at 600 nm) after incubation. (B) Bright-field (upper) and fluorescence microscopy (lower) images of BSA-RITC-encapsulated PC@MPNs. (C) Zeta potential of PC@MPNs and PC-IFNα@MPNs. (D) Young's modulus of PC@MPNs and PC-IFNα@MPNs. (E) Turbidity of PC@MPNs after incubation in 100% FBS at 37 °C for 24 h.

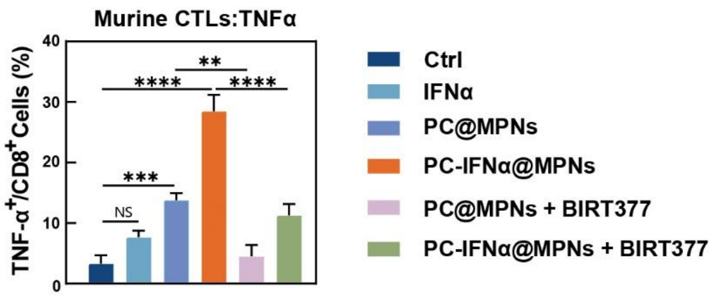

Figure S4. Flow cytometry detection of intracellular staining of TNF-α^+ cells in $CD8^+$ cells.

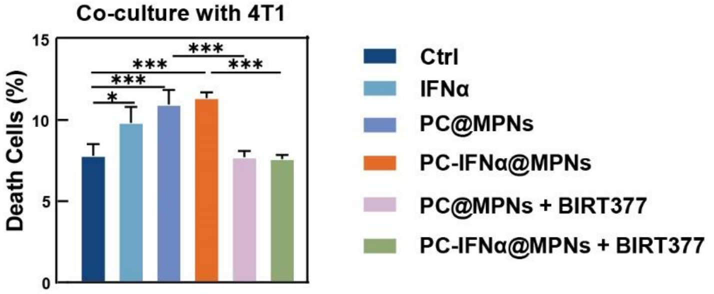

Figure S5. 4T1-OVA cells labeled with CFSE were cocultured with CD8$^+$ T cells isolated from the spleen of OT-1 mice at a CD8$^+$ T cell-to-4T-1-OVA cell ratio of 1:5 under different treatments for 24 h. Samples were stained with 7-AAD and analyzed using flow cytometry.

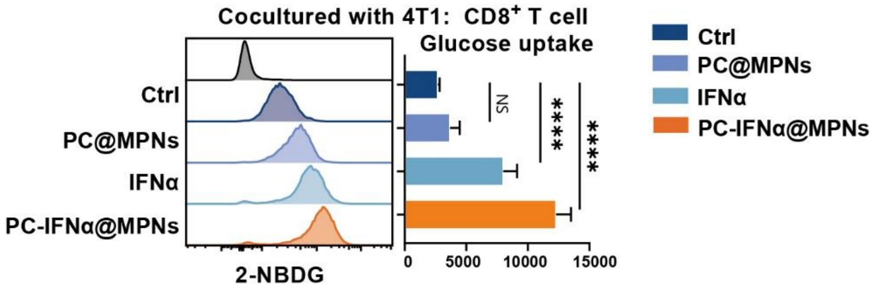

Figure S6. 2-NBDG uptake of CD8$^+$ T cells cocultured with 4T1 cells.

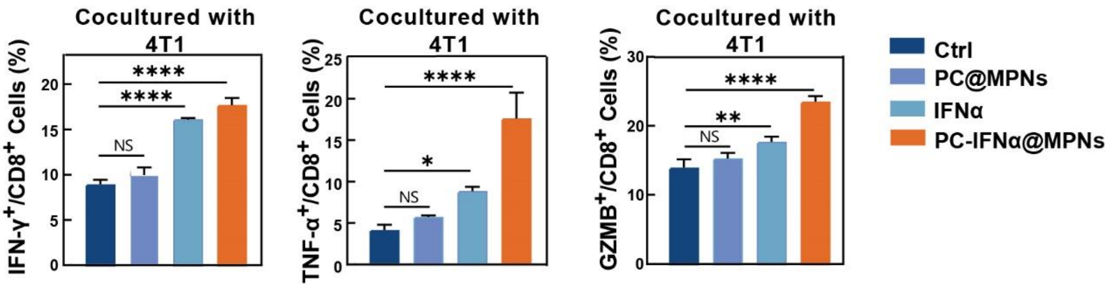

Figure S7. Intracellular staining of IFN-γ$^+$, TNF-α$^+$, and GZMB$^+$ cells in CD8$^+$ T cells cocultured with 4T1 cells.

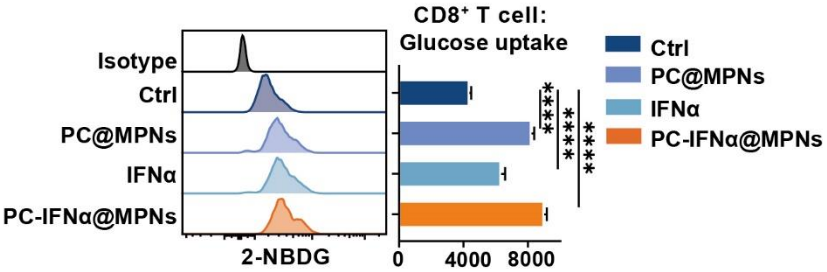

Figure S8. 2-NBDG uptake of $CD8^+$ T cells treated with PBS, PC@MPNs, IFNα, or PC-IFNα@MPNs.

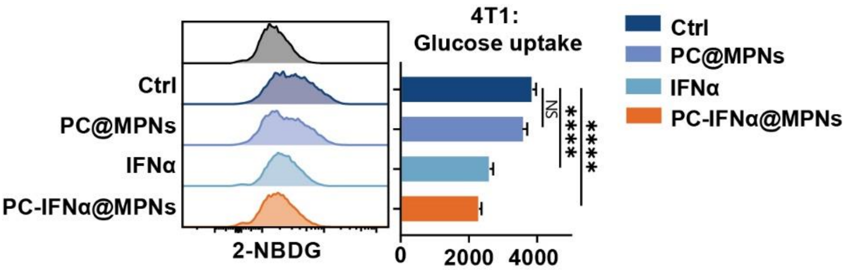

Figure S9. 2-NBDG uptake of 4T1 cells in coculture system.

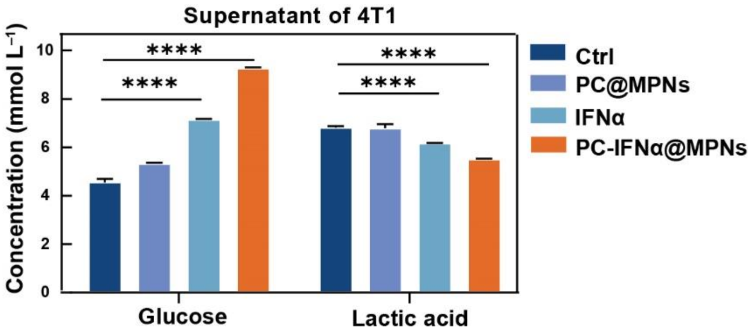

Figure S10. Concentrations of glucose and lactic acid in cellular supernatants of 4T1 after different treatments for 24 h.

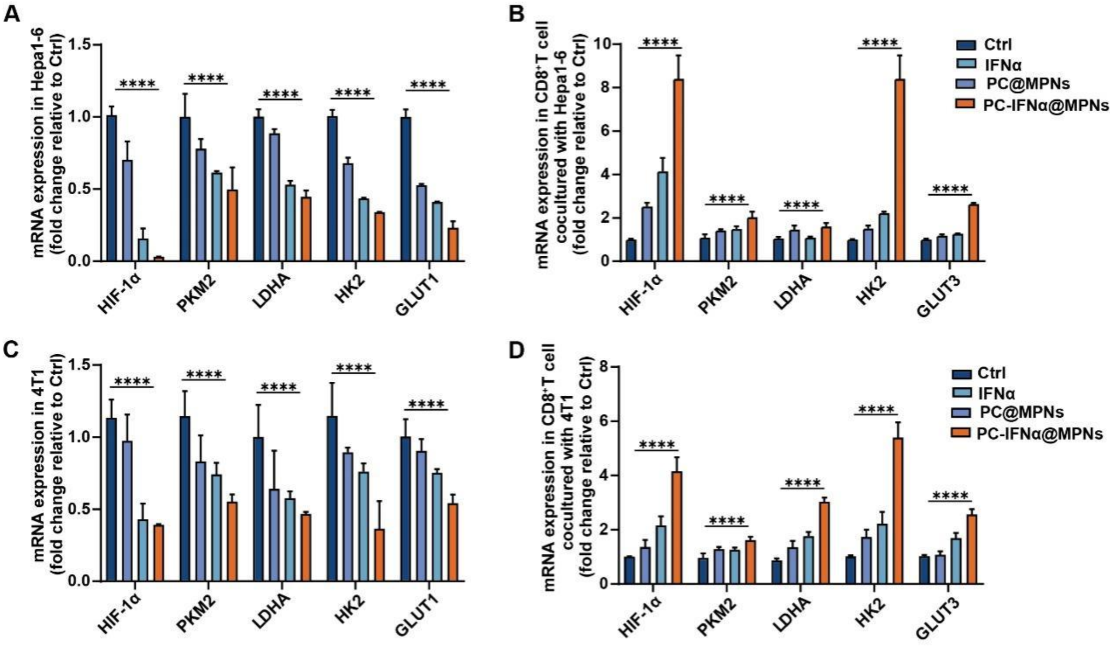

Figure S11. (A–D) Quantitative reverse-transcription PCR validation of glycolysis genes in different treatment groups in Hepa1-6 (A), CD8$^+$ T (B), 4T1 (C), and CD8$^+$ T (D) cells.

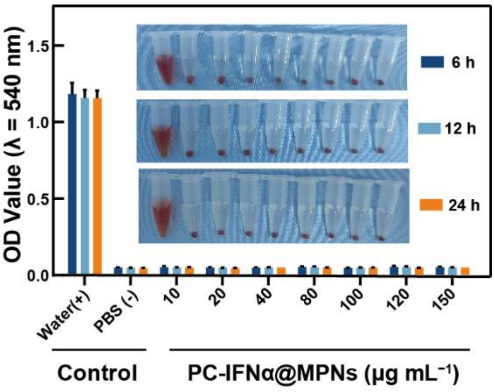

Figure S12. Detection of hemolysis based on optical density (OD) measurements at different time points after in vitro treatment of mouse blood with different doses of PC-IFNα@MPNs. (+): Positive control, (−): negative control.

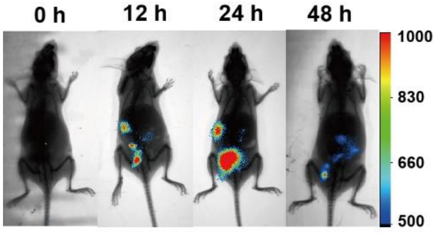

Figure S13. In vivo imaging at different time points after injecting DiR-labeled PC-IFNα@MPNs into the tail vein of orthotopic BC model mice.

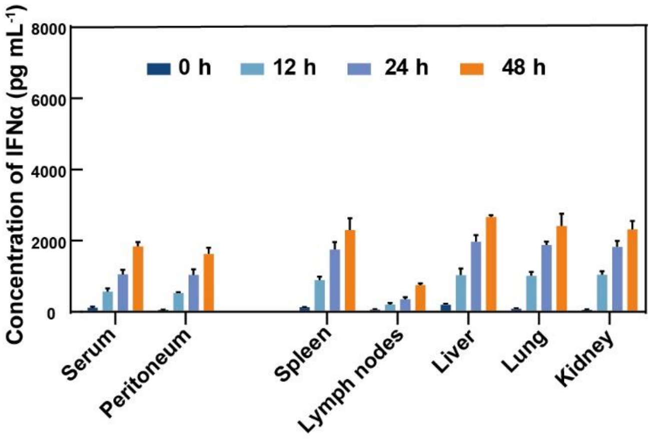

Figure S14. IFNα levels in serum and interorgan fluid of orthotopic HCC model mice after intravenous injection with PC-IFNα@MPNs (20 mg kg^{-1}).

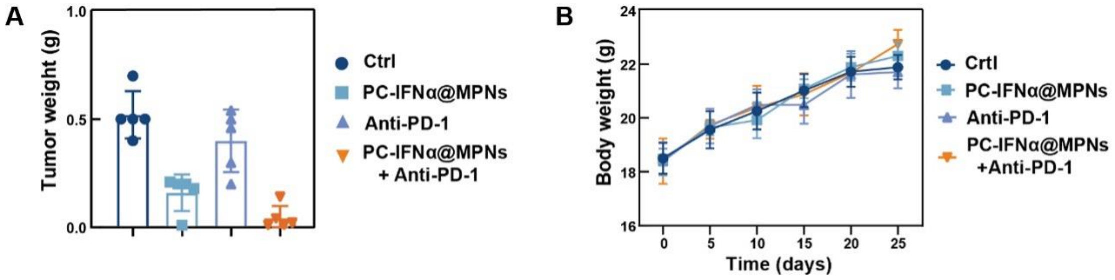

Figure S15. (A) Weight of tumor used in the different treatment groups. (B) Changes in body weight of orthotopic HCC model mice subjected to different treatments.

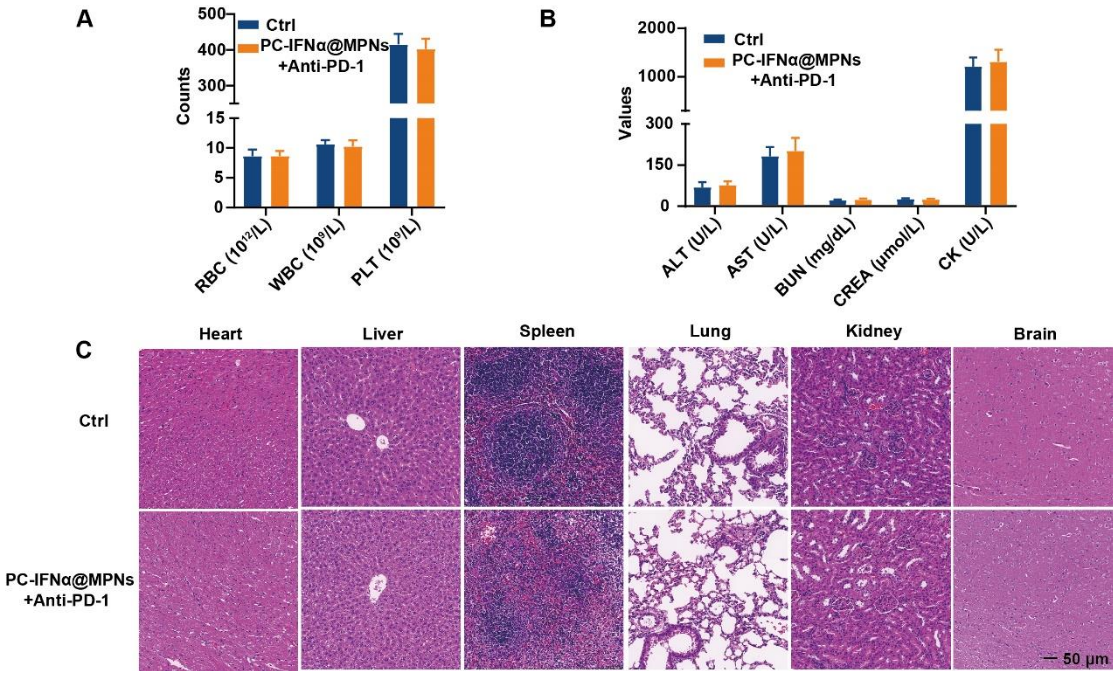

Figure S16. (A, B) Analysis of complete blood count (A) and serum biochemical molecules (B) in mice of orthotopic HCC model. RBC: red blood cell; WBC: white blood cell; PLT: platelet; ALT: alanine aminotransferase; AST: aspartate aminotransferase; BUN: blood urea nitrogen; CREA: creatinine; CK: creatine kinase. (C) Representative histological examination of the heart, liver, spleen, lung, kidney, and brain of mice of orthotopic BC model using hematoxylin & eosin staining. Scale bar for all images is 50 µm.

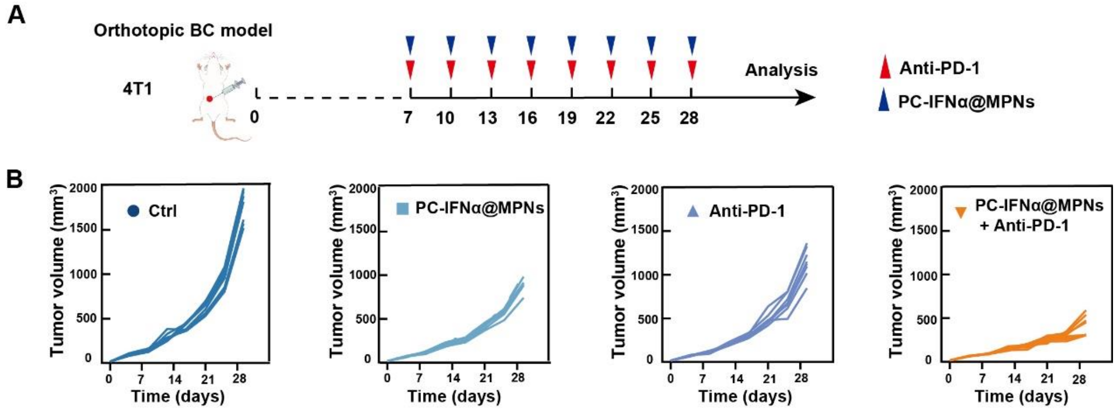

Figure S17. (A) Schematic of experimental design of orthotopic BC model. (B) Tumor volume profiles of mice subjected to different treatments.

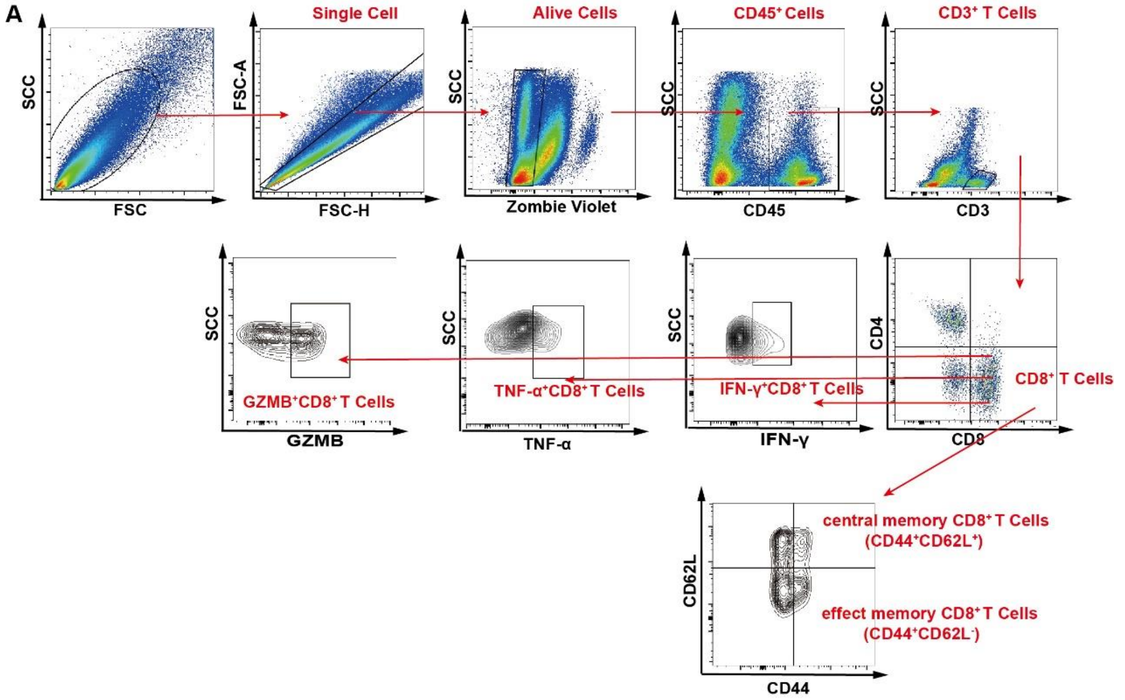

Figure S18. Gating strategy in FACS.

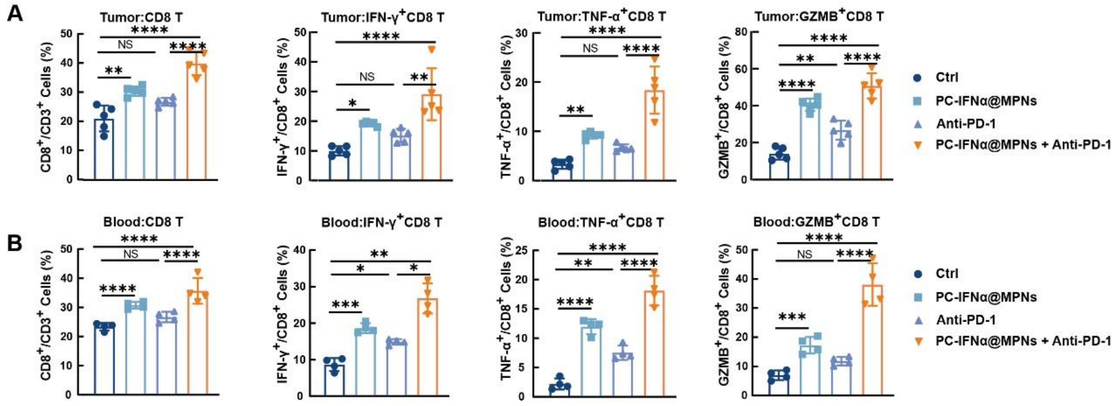

Figure S19. (A, B) Percentages of CD8^{+} T cells, and IFN-γ^{+}, TNF-α^{+}, and GZMB^{+} CD8^{+} T cells in tumor tissue (A) and blood (B) of orthotopic BC model, as measured by flow cytometry.

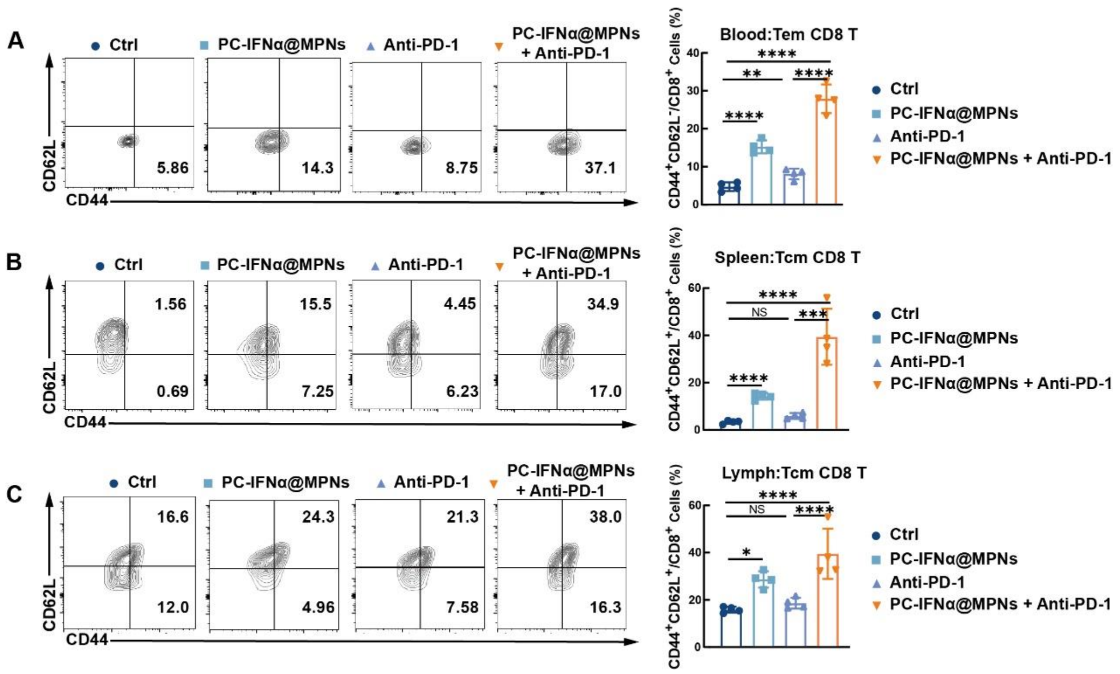

Figure S20. (A) Representative flow cytometry analysis images (left) and relative quantification (right) of CD44^{+} CD62L^{-} Tem cell gating on CD8^{+} T cells in blood and of orthotopic BC model. (B, C) Representative flow cytometry analysis images (left) and relative quantification (right) of CD44^{+}CD62L^{+} Tcm cells gating on CD8^{+} T cells in the spleen (A) and inguinal lymph node (C) of orthotopic BC model.

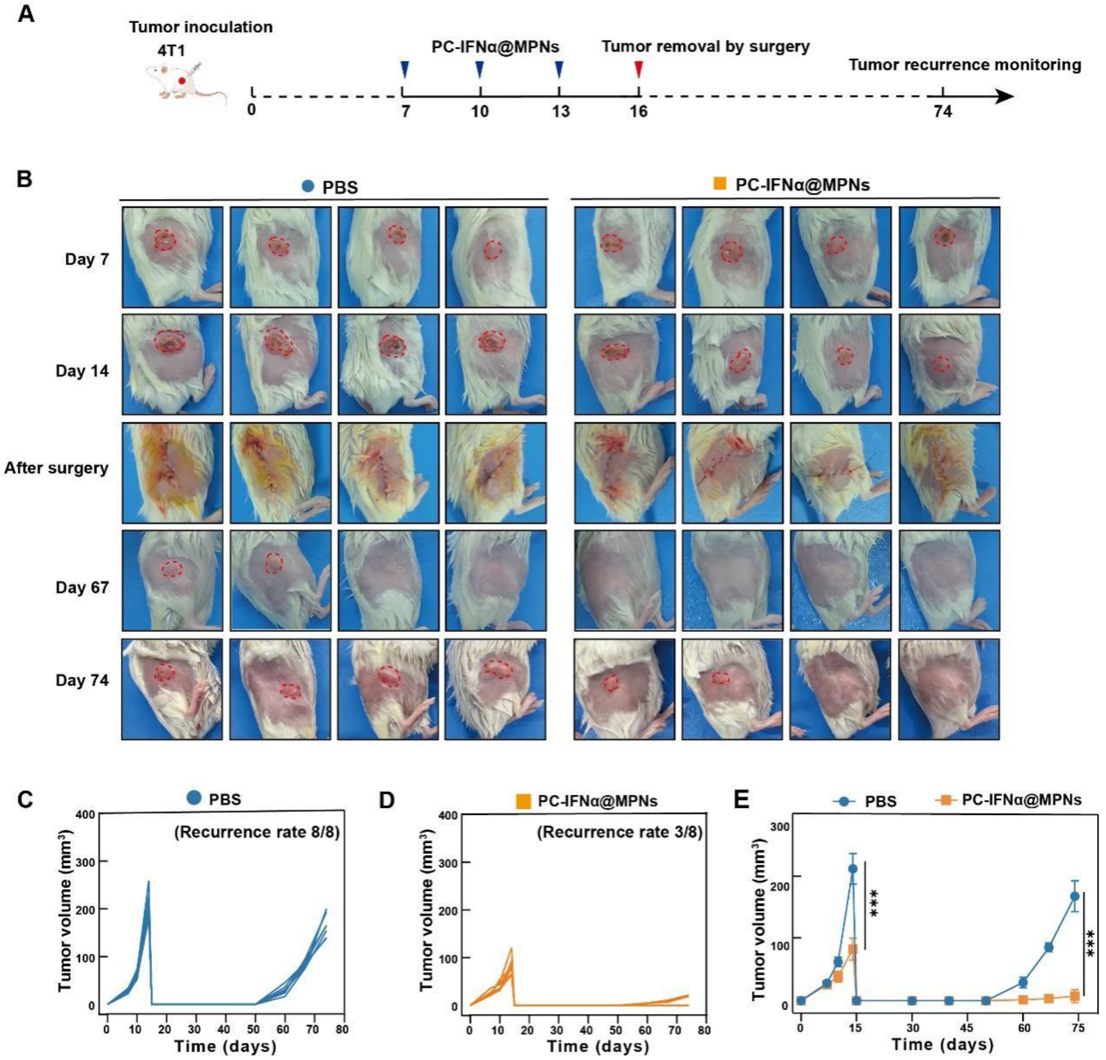

Figure S21. (A) Timeline of study conducted using the 4T1 subcutaneous incomplete resection tumor model. Intravenous injection of PBS or PC-IFNα@MPNs was performed on days 7, 10, and 13 after tumor implantation. Surgical resection of tumor was performed on day 16. (B) Representative photographs of mice. (C, D) Tumor volume profiles of mice subjected to different treatments: PBS (C) and PC-IFNα@MPNs (D). (E) Primary tumor growth curves.
